# Mitochondrial Oxidative Stress, Mitochondrial DNA Damage and Their Role in Age-Related Vascular Dysfunction

**DOI:** 10.3390/ijms160715918

**Published:** 2015-07-13

**Authors:** Yuliya Mikhed, Andreas Daiber, Sebastian Steven

**Affiliations:** 1 2nd Medical Clinic, Medical Center of the Johannes Gutenberg-University, Mainz 55131, Germany; E-Mails: yuliya.mikhed@unimedizin-mainz.de (Y.M.); sebastiansteven@gmx.de (S.S.); 2 Center for Thrombosis and Hemostasis, Medical Center of the Johannes Gutenberg-University, Mainz 55131, Germany

**Keywords:** aging, mitochondrial oxidative stress, mitochondrial DNA damage, vascular dysfunction

## Abstract

The prevalence of cardiovascular diseases is significantly increased in the older population. Risk factors and predictors of future cardiovascular events such as hypertension, atherosclerosis, or diabetes are observed with higher frequency in elderly individuals. A major determinant of vascular aging is endothelial dysfunction, characterized by impaired endothelium-dependent signaling processes. Increased production of reactive oxygen species (ROS) leads to oxidative stress, loss of nitric oxide (^•^NO) signaling, loss of endothelial barrier function and infiltration of leukocytes to the vascular wall, explaining the low-grade inflammation characteristic for the aged vasculature. We here discuss the importance of different sources of ROS for vascular aging and their contribution to the increased cardiovascular risk in the elderly population with special emphasis on mitochondrial ROS formation and oxidative damage of mitochondrial DNA. Also the interaction (crosstalk) of mitochondria with nicotinamide adenosine dinucleotide phosphate (NADPH) oxidases is highlighted. Current concepts of vascular aging, consequences for the development of cardiovascular events and the particular role of ROS are evaluated on the basis of cell culture experiments, animal studies and clinical trials. Present data point to a more important role of oxidative stress for the maximal healthspan (healthy aging) than for the maximal lifespan.

## 1. Introduction

**Figure 1:**
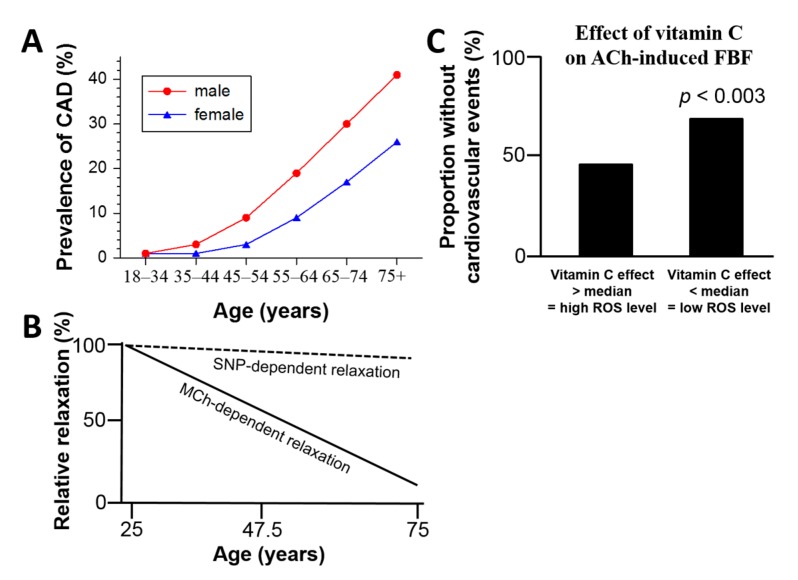
(**A**) Prevalence of coronary artery diseases (CAD) increases with progressing age and gender in Germany. Drawn from results of the Detect Study [26,27] and figure adopted from [28] with permission of the publisher. © Springer Science+Business Media, LLC 2010; (**B**) Correlation between age and endothelium-dependent (methacholine (MCh), solid line) and -independent (sodium nitroprusside (SNP), broken line) relaxation. Healthy individuals with an age of 25–70 years were tested for MCh- and SNP-dependent vasodilation. Endothelium-dependent relaxation was impaired with age (*r* = 0.81, *p* < 0.001, *r* is the correlation coefficient), whereas endothelium-independent relaxation was decreased only by trend in older individuals (*r* = 0.1, not significant). According to [24]; and (**C**) Results from Kaplan-Meier-analysis for the cardiovascular event rate in two cohorts of patients displaying either pronounced or weak effect of vitamin C on endothelial function (measured by forearm plethysmography after infusion of acetylcholine (ACh)) over a time period of more than 6 years. The take-home message is: Higher levels of vascular oxidative stress (free radicals) are associated with a more pronounced beneficial effect of the radical scavenger vitamin C on endothelial function and an increased cardiovascular event rate. FBF means forearm blood flow. According to Heitzer *et al.*, Circulation 2001 [25].

Demographic change is an emerging issue in the Western world. The proportion of people older than 65 years will dramatically increase within the next decades [1]. Besides its negative effects on the costs for retirement funds, an increasing average age will amplify the economic burden for healthcare costs in these countries. Cardiovascular diseases (CVD) are a main cause of morbidity and mortality in elderly people and their incidence is closely correlated with age [2] (Figure 1A). The term “vascular aging” outlines all changes in vessels, which are associated with aging. Smooth muscle cells and endothelial cells are involved in these changes during vascular aging. Progressive aging leads to arterial stiffness and endothelial dysfunction, which is known to correlate with future cardiovascular events in humans [3]. Furthermore, aged vessels are more prone to develop atherosclerotic lesions, vascular injury, impaired angiogenesis and calcification [4]. Consequently, the incidence and frequency of cardiovascular diseases such as atherosclerosis and its late complications such as coronary artery disease or stroke, increase substantially with age [5]. However, endothelial dysfunction in the elderly is not only associated with CVD, but also with other disorders related to aging, such as erectile dysfunction, renal dysfunction, Alzheimer’s disease or retinopathy [6,7,8,9]. Since the CVD burden is predicted to increase dramatically in Western societies and the knowledge about vascular aging is limited, there is urgent need for research in this field in order to reduce morbidity and mortality in the aging population. Within the last years, scientists identified three key players in the vascular aging process: nitric oxide signaling, oxidative stress and inflammation [10]. It should be noted, that these players do not stand alone as they affect and influence each other. Especially pathophysiological convergence of different organ diseases with associated comorbidities increases at higher age and represents another important field of research that needs to be addressed in the future [11,12,13].

Nitric oxide (^•^NO) is essential for a functional endothelium and diminished ^•^NO bioavailability leads to endothelial dysfunction [14,15]. In aged vessels the bioavailability of ^•^NO is reduced, whereas production of reactive oxygen species (ROS) is increased [10,16,17]. It is not only the reaction of ^•^NO with superoxide anion (O_2_^•−^), leading in turn to production of peroxynitrite (ONOO^−^) [17], which reduces ^•^NO bioavailability. Also dysregulation of the endothelial nitric oxide synthase (eNOS), known as eNOS uncoupling, results in impaired ^•^NO release from the endothelium and leads instead to increased superoxide production [18]. The mechanisms of this uncoupling process are complex and multiple. They include decreased availability of the eNOS substrate l-arginine or the cofactor tetrahydrobiopterin (BH4), but also phosphorylation state (Ser1177, Thr495, Tyr657) or *S*-glutathionlyation of the protein (for review see [19]). All of them play an important role for the coupling state of the enzyme and many of them were identified in the vascular aging process [16,20,21,22]. Imbalance of ^•^NO bioavailability by ROS is not only induced by eNOS itself. Increased oxidative stress from mitochondria and other enzymatic sources are observed in aged animals and affect the coupling state of eNOS [23]. This observation points to a strong correlation between aging, oxidative stress, and as a consequence of imbalanced ^•^NO bioavailability, the development of endothelial dysfunction (Figure 1B) [24]. The impact of vascular oxidative stress on endothelial function and the predictive value of this parameter was previously shown by a large clinical trial demonstrating better cardiovascular prognosis in patients with lower burden of vascular oxidative stress (less pronounced effect of vitamin C infusion on flow-mediated dilation) (Figure 1C) [25].

There is good evidence that mitochondria represent a major source of ROS in aging tissues [29,30]. Mitochondrial DNA damage accumulates in the aging cell leading to mitochondrial dysfunction [31] and aging-related cardiovascular and neurodegenerative disease [10,32]. The present review will discuss the impact of mitochondrial oxidative stress and mitochondrial DNA damage on vascular dysfunction in the aging process.

## 2. The Cardiovascular System

The cardiovascular system is a closed network containing arteries, veins and capillaries. The center of this network is the heart. Transportation is one of the most important functions of the human cardiovascular system. By every heartbeat nutrients, oxygen, carbon dioxide and hormones are distributed to all parts of the body. Furthermore, the cardiovascular system is involved in host defense by the inflammatory system and hemostasis by the coagulation system. As the interface between blood and vessel wall, the endothelium plays a crucial role as a specialized monolayered squamous epithelium that lines the interior surface of blood vessels. Preserving the blood barrier function and thereby preventing adhesion of immune cells is a defense against infiltration of immune cells such as monocytes into lesion-prone areas of the endothelium, an essential step in the development of atherosclerotic plaques [33,34]. There are over 2 billion heartbeats in one human life and every heartbeat is associated with increased sheer stress and elongation of the vessel. The endothelial cell layer has to control the vascular tone under all circumstances by nitric oxide (^•^NO), endothelium-derived hyperpolarizing factor, prostacyclin or natriuretic peptides. Furthermore, these mediators released by the endothelium have antiaggregatory properties and suppress thrombus formation, vascular stenosis [35] and cardiac hypertrophy. On the other hand, the endothelium acts synergistically with a regulatory system, which consists of vasoconstrictors such as catecholamines and other vasoactive peptides (*i.e.*, angiotensin, vasopressin, endothelin).

The aging endothelium is more and more unable to fulfill all these tasks. In elderly people a significant impairment of endothelium-dependent relaxation (endothelial dysfunction) can be found [36,37]. This dysfunction promotes thrombosis, vasoconstriction, leukocyte infiltration and cell proliferation in the vessel wall. Thus, endothelial dysfunction in aging is an early predictor for the development of atherosclerosis, hypertension and future cardiovascular events. Besides this interaction during the aging process, this correlation was also proven by a meta-analysis of 23 studies [3], which nicely demonstrates flow-mediated dilation (FMD) of the brachial artery as a prognostic marker for cardiovascular events. Although this study cannot prove endothelial dysfunction as the cause of increased cardiovascular morbidity, it demonstrates that endothelial dysfunction is a precursor of cardiovascular disease. Not only are macrovessels, like aorta or coronary arteries, affected by aging-dependent endothelial dysfunction and oxidative stress but the microcirculation (resistance vessels) are especially affected by vascular aging (for review see [38]). Studies of Mayhan *et al.* demonstrated impaired eNOS-dependent reactivity of cerebral arterioles, which was associated with increased oxidative stress [39]. Similar evidence for endothelial dysfunction could be found for retinal vessels during the aging process [40] and its contribution to neurodegenerative disease is very likely [41,42] Our group and many others revealed impaired ^•^NO signaling, vascular inflammation and oxidative stress as key players in the pathogenesis of aging dependent endothelial dysfunction (for review see [28]).

## 3. Aging and Oxidative Stress

As early as in 1954, Harman expressed for the first time the free radical theory of aging [43]. This idea was based on the observations, that aging is a universal phenomenon, and its contributing factors must be present in every living organism. His first hypothesis emphasized the importance of the hydroxyl radical, as well as molecular oxygen in the aging process [44]. Later, this concept was extended to mitochondria which are the most abundant cellular source of ROS. Mitochondrial ROS formation probably contributes to the high mutation rate of the mitochondrial genome. In general, assembly of the respiratory chain components requires the contribution of two spatially separated genomes, the nuclear DNA and the maternally inherited mtDNA [45]. Malfunctioning of the mitochondrial genome is directly correlated with impaired mitochondrial physiology and depleted ATP-synthesis, which are accompanied by enhanced ROS formation and increased apoptosis [29]. Age-dependent impairment of vascular redox regulation is demonstrated by the bioavailability of another free radical species –^•^NO. Nitric oxide is not only involved in vasodilation, but also in vascular smooth muscle cell proliferation, inhibition of platelet aggregation and several others [46]. It has been postulated that ^•^NO is gradually reduced with age and might serve as an applicable biomarker for age-dependent endothelial dysfunction. The prevailing paradigm is that an age-dependent increase in superoxide rapidly consumes ^•^NO, consequently reduces its endothelial levels and thereby leads to impaired vasorelaxation [24,47].

Oxidative stress burden usually correlates with cellular thiol levels or vice versa cellular thiol/disulfide ratio is a well-accepted indicator of the redox state of a cell. Therefore, thiols and thiol-dependent enzymes were in the focus of oxidative stress and aging-related research. Cellular thiols possess significant antioxidant effects and affect the organismal healthspan. Glutathione peroxidases (GPx) belong to the class of enzymes responsible for the removal of H_2_O_2_ from the intracellular compartments. *GPx* deficiency leads to increased levels of oxidative stress, pronounced vascular dysfunction [16] and increased senescence of fibroblasts [48]. Even though genetic depletion of either *GPx-1* or *GPx-4* has no effect on the lifespan of the experimental animals, their effect on the process of healthy aging cannot be disputed [16]. Thioredoxins (Trx) are another class of antioxidant enzymes that can directly react with peroxides and eliminate damage caused by peroxides via reduction of disulfides and methionine sulfoxides [49]. Complete knock-out of the mitochondrial Trx isoform (*Trx2^−/−^*) is embryonically lethal and partial knock-out (*Trx2^+/−^*) mice show elevated levels of lipid peroxides, oxidized nucleobases and proteins [50]. Although *Trx2^+/−^* mice exhibited reduction in their lifespan by trend, a further increase of the significance power would require higher number of animals. On the other hand, genetic knock-in of the cytosolic isoform of thioredoxin, *Trx1*, showed considerable increase in mice longevity and stronger resistance to oxidative stress inducers, like UV-light or ischemia/reperfusion, further supporting the previous notion of the importance of antioxidant enzymes [51].

The impact of antioxidant defense enzymes on aging-related cardiovascular complications is well established and has been previously demonstrated for the mitochondrial superoxide dismutase 2 (SOD2) [52], the cytosolic superoxide dismutase (SOD1) [47,53], the extracellular superoxide dismutase (ecSOD), and the thioredoxin-1 protein (Trx) [49]. Considering the fact that superoxide is the major contributing factor to the endothelial dysfunction in the aging vasculature further investigations of the antioxidant systems have been conducted in order to understand why these defense mechanisms are not able to combat increasing levels of the oxidative stress. On the example of SOD2, it has been shown that in aging vessels, MnSOD has been heavily nitrated, most probably by peroxynitrite, as indicated by increased staining for 3-nitrotyrosine [54]. Inhibition of this protective enzyme results in the activation of the vicious cycle of increased oxidative burden. On the other hand, no direct correlation between lifespan and deletion of or overexpression of most antioxidant enzymes (*SOD2^+/−^* or transgenic overexpression of *SOD2* (*SOD2^tg^*), *GPx-1^−/−^*, *GPx-4^−/−^* or *MsrA^−/−^*, transgenic overexpression of *SOD1* (*SOD1^tg^*), transgenic overexpression of catalase (*catalase^tg^*)) could affect the longevity [55]. Only *SOD1^−/−^* mice and mice with double gene ablation combinations showed reduced life expectancy [55,56]. It is worth to mention that mice completely deficient in *SOD2* show lethality at the embryo stage or a few weeks after birth, once again stressing the importance of these antioxidant enzymes in the normal physiology [57,58]. Of note, overexpression of *Trx1* increased the lifespan and stress resistance [51]. Although there is only a limited role for oxidative stress as a direct determinant for accelerated aging or decreased lifespan [55,56,59], there is substantial evidence for a contribution of oxidative stress to detrimental effects on physiological organ function during the aging process preventing healthy aging [60,61,62,63].

The observation that antioxidant enzymes have a significant effect on the healthspan of animals during normal aging (e.g., indicated by decreased aging-associated cardiovascular complications during the sunset years) and also on the resistance to stress conditions is of high clinical importance [51]. Previously, we demonstrated that genetic deletion of the mitochondrial antioxidant proteins aldehyde dehydrogenase-2 (*ALDH-2*) and manganese superoxide dismutase (*Mn-SOD*) leads to vascular dysfunction and mitochondrial oxidative stress with increasing age [23]. These data support the concept that oxidative stress in general and mitochondrial ROS formation in particular, despite not playing a key role for the lifespan, have significant impact on the quality of aging, the healthspan [60,61,62,63].

## 4. Vascular Function, Oxidative Stress and ^•^NO Bioavailability in Aging

Endothelial dysfunction was found in several animal models of hypertension or atherosclerosis, both representing important cardiovascular risk factors (for review see [19]). Furthermore, patients with endothelial dysfunction display a higher burden of oxidative stress and have increased risk factors for cardiovascular disease and events (see Figure 1). Endothelial dysfunction and most cardiovascular disease are characterized by increased levels of ROS formation due to an imbalance between pro-oxidative enzymes (xanthine oxidase, NADPH oxidase, uncoupled eNOS or enzymes of mitochondrial respiration) and antioxidant enzymes (Cu, Zn-SOD, Mn-SOD and extracellular SOD), resulting in a deviation of cellular redox environment from the normal [64]. A similar pattern of vascular dysregulation can be found in aging tissues (for review see [28]) and was first described in 1956 by Harman *et al.* (“free radical theory of aging”).

Irreversible oxidations and accumulation of oxidized biological macromolecules (e.g., DNA mutations, oxidized proteins) appear in biological systems, which are suffering from chronic oxidative stress. Besides these long-term consequences, ROS interfere rapidly with nitric oxide (^•^NO) signaling. The accepted concept for reduced ^•^NO bioavailability is the reaction of superoxide with ^•^NO under formation of peroxynitrite (ONOO^−^) [65]. Not only is reduced bioavailability of the important vasodilator ^•^NO problematic for the vascular system, ONOO^−^ itself has the ability to disturb the enzymatic function of proteins by nitration of tyrosine residues and oxidation of cysteine-thiol-groups [66,67,68]. Among others, the mitochondrial isoform of superoxide dismutase, Mn-SOD, is affected by nitration and becomes inactivated which further reduces antioxidant capacity of the cell [69].

The ^•^NO producing enzyme eNOS itself is highly susceptible to damage by increased oxidative stress [15]. Tetrahydrobiopterin (BH4), a cofactor of eNOS, can be oxidized by ONOO^−^ and the latter can potentially uncouple eNOS [70,71]. BH4 is a redox cofactor of eNOS and regulates the catalytic activity. In aged animals reduced vascular BH4 levels were shown [72], but the results in the literature are controversial [73]. Nevertheless, pharmacological supplementation of BH4 improves endothelial function in aged humans compared to young subjects [22]. This shortage of cofactor leads to a conformational change in eNOS resulting in uncoupling. Besides BH4, eNOS has several other redox switches that may lead to dysfunction/uncoupling in a ROS-dependent fashion: it can be *S*-glutathionylated [74], inhibited by asymmetric dimethylarginine (ADMA) and phosphorylated in a protein kinase C (PKC) or protein tyrosine kinase (PYK-2) dependent manner at Thr495 or Tyr657 [75]. Likewise, the zinc-sulfur-complex at the dimer-binding-interface can be oxidatively disrupted [76].

Uncoupling of eNOS switches the enzyme from good to evil [77]. In the uncoupled state, eNOS is generating ROS, which further oxidize BH4 and reduce ^•^NO bioavailability [71]. This vicious circle is an established concept and part of the pathogenesis of endothelial dysfunction in aged vessels [24,36]. Several groups reported on increased eNOS expression levels in aging, which might be a counter-regulatory effect to compensate for decreased ^•^NO bioavailability. In contrast, other groups found no change of eNOS expression in aging, but observed decreased Akt-dependent phosphorylation of eNOS at Ser1177 with increasing age as a potential explanation for an impaired endothelial dysfunction in the elderly [78]. Our group just recently provided evidence for *S*-glutathionylation and adverse phosphorylation of eNOS at Thr495 and Tyr657 by redox-sensitive PKC and PYK-2, respectively, as important mechanisms in the process of aging-induced vascular dysfunction [16].

NADPH oxidase (Nox) is a major source of ROS in the cardiovascular system [79,80]. Isoforms 1, 2, 4 and 5 are significantly expressed in heart and vessels. Nox2 and Nox4 are known to be upregulated in vascular tissue of aged mice [81]. In addition, these enzymes are regulated by tumor necrosis factor-α (TNF-α), which is known to be elevated in aged animals and humans [82,83]. The cytokine TNF-α plays an important role in many inflammatory disorders and vascular dysfunction is closely linked to inflammatory processes [84]. In fact, administration of TNF-α can promote oxidative stress by activation of Nox, endothelial dysfunction, endothelial apoptosis, and upregulation of proatherogenic inflammatory mediators, like inducible nitric oxide synthase (iNOS) and adhesion molecules [85,86]. Furthermore, TNF-α stimulates mitochondrial superoxide production in human retinal endothelial cells [87]. Chronic TNF-α inhibition improves flow-mediated arterial dilation in resistance arteries of aged animals, while reducing iNOS and intercellular adhesion molecule-1 (ICAM-1) expression [88]. All the mentioned effects are similar to functional alterations of the aged vascular endothelium. Not only TNF-α, but also interleukins (IL-1β, IL-6, IL-17) and C-reactive protein (CRP) are elevated in aging independent to other risk factors (e.g., smoking) [89]. Since, infiltrating leukocytes contribute to increased oxidative stress and reduced ^•^NO bioavailability in the vessel wall [90,91], cytokine release and chemoattraction of leukocytes by the endothelium are important in the pathogenesis of aging-mediated endothelial dysfunction.

## 5. Aging, Mitochondrial Oxidative Stress, Mitochondrial DNA Damage and Endothelial Dysfunction

In 1972, Harman modified his “free radical theory of aging” to specify the role of mitochondria [92]. He tried to explain why exogenous supplementation of antioxidants to rodents could not improve their lifespan. His explanation was that these antioxidants do not reach the mitochondrion. He proposed that mitochondria are both the primary origin and target of oxidative stress.

**Figure 2:**
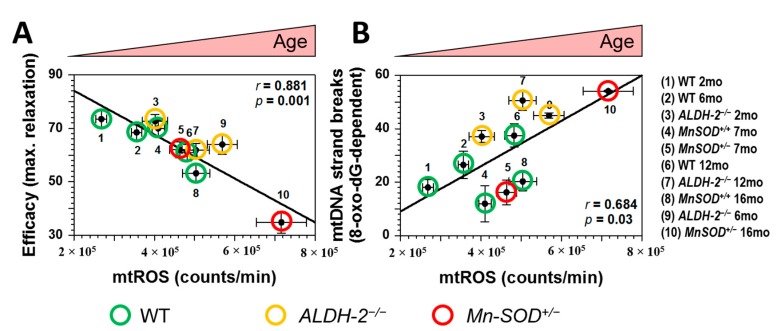
Correlations between mitochondrial oxidative stress (mtROS), mitochondrial DNA (mtDNA) damage and vascular (endothelial) function (ACh-induced maximal relaxation). (**A**) mtROS formation was plotted for all age-groups and mouse strains *versus* the corresponding maximal efficacy in response to acetylcholine (ACh); (**B**) mtROS was plotted for all age-groups and mouse strains *versus* the corresponding mtDNA damage. ROS were measured using L-012 (100 µM) enhanced chemiluminescence in isolated cardiac mitochondria upon stimulation with succinate (5 mM). *r* is the correlation coefficient. The groups are: 1 = B6 WT, 2mo; 2 = B6 WT, 6mo; 3 = *ALDH-2^−/−^*, 2mo; 4 = *MnSOD^+/+^*, 7mo; 5 = *MnSOD^+/−^*, 7mo; 6 = WT B6, 12mo; 7 = *ALDH-2^−/−^*, 12mo; 8 = *MnSOD^+/+^*, 16mo; 9 = *ALDH-2^−/−^*, 6mo; 10 = *MnSOD^+/−^*, 16mo. The age of measured groups increases from the left to the right. Adopted from Wenzel *et al.*, Cardiovasc. Res*.* 2008 [23]. With permission of the European Society of Cardiology. All rights reserved. © The Author and Oxford University Press 2008.

Recently, we demonstrated in two different knock-out models with increased mitochondrial ROS (*ALDH-2^−/−^*, *MnSOD^−/−^* mice), that mitochondrial ROS formation and oxidative mitochondrial DNA (mtDNA) lesions as well as vascular dysfunction are increasing with age [23] (Figure 2). According to our data, endothelial dysfunction was clearly correlated with mitochondrial oxidative stress. The increase of mitochondrial ROS was more dependent on aging, then on the presence or absence of antioxidant proteins. The correlation between mtROS and mtDNA strand breaks, led us to speculate that the mitochondrial DNA damage could induce even more mtROS. Since the mitochondrial DNA mainly encodes for proteins of the mitochondrial respiration chain, one could assume that impaired mtDNA translation leads to mitochondrial uncoupling with secondary increase in mtROS formation.

Previous reports highlighted increased mitochondrial and systemic oxidative stress in mice with genetic deficiency in glutathione peroxidase-1 (*GPx-1*) [48]. In addition, *GPx-1* deficiency showed synergistic negative effects on vascular function in the setting of diabetes [93], hyperlipidemia [94], and hypertension [95]. Moreover, increased senescence was reported for fibroblasts from *GPx-1^−/−^* mice [48]. Most importantly, a correlation between cardiovascular risk and GPx-1 activity in blood cells was previously reported conferring high clinical relevance to the expression/activity of GPx-1 [96] and again supporting the concept that antioxidant enzymes and oxidative stress might contribute significantly to the healthspan and comorbidity of the elderly [60,61,62,63].

**Figure 3:**
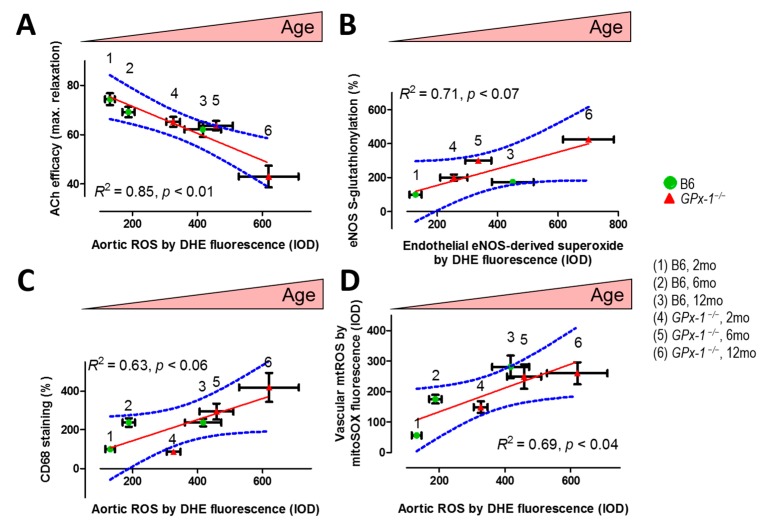
(**A**) Correlation between endothelium (ACh)-dependent relaxation (isometric tension measurement in isolated aortic ring segments) and aortic ROS formation (DHE staining of the aortic wall). Linear regression: *p* < 0.01, *R*^2^ = 0.85; (**B**) Correlation between eNOS uncoupling marker (*S*-glutathionylated eNOS) and endothelial (eNOS-derived) superoxide formation (endothelial DHE staining). Linear regression: *p* < 0.07, *R*^2^ = 0.71; (**C**) Correlation between inflammation (CD68 staining) and aortic ROS formation (DHE staining of the aortic wall). Linear regression: *p* < 0.06, *R*^2^ = 0.63; and (**D**) Correlation between mitochondrial ROS formation (mitoSOX staining) and aortic ROS formation (DHE staining of the aortic wall). Linear regression: *p* < 0.04, *R*^2^ = 0.69. Linear regressions were performed with GraphPad Prism 6 for Windows (version 6.02). All data were collected from our previous work [16]. Each data point was based on measurement of 4–10 animals. B6 means C57/BL6 wild type control; *GPx-1^−/−^* means glutathione peroxidase-1 knockout mice on C57/BL6 background. The age of measured groups increases from the left to the right. The solid red lines are simple linear regression fits to the data. Blue lines are the 95% confidence intervals on the estimated means. With permission of Wolters Kluwer Health, Inc. Copyright © 2014, Wolters Kluwer Health.

**Figure 4:**
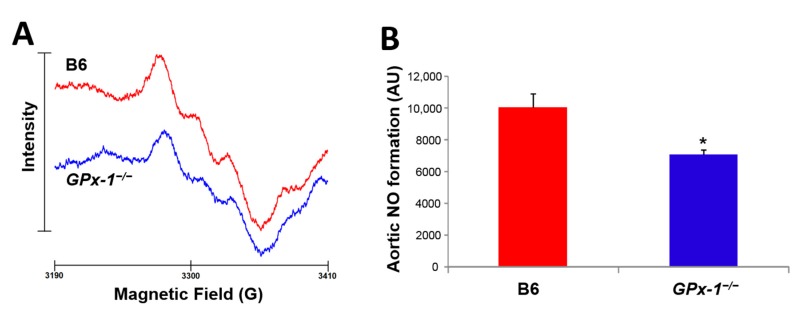
Nitric oxide formation in isolated aortic ring segments in old (12 mo) wild type (WT) and *GPx-1* knockout mice. ^•^NO was measured in aortic ring segments (1 thoracic aorta for each measurement) upon stimulation with calcium ionophore (A23187, 10 µM) for 60 min at 37 °C in the presence of freshly prepared lipophilic spin trap Fe(II)(DETC)_2_. ^•^NO bound to the spin trap generates a stable paramagnetic nitrosyl-iron species that yield a typical triplet signal when measured by electron spin resonance (EPR) spectroscopy in liquid nitrogen. The detailed method was published in [97,98] and samples were used from a published study [16]. (**A**) Representative spectra and (**B**) quantification of signal intensity. Data are mean ± SEM of 9 mice per group. *, *p* < 0.05 *versus* wild type.

Recently, we demonstrated for the first time that aging per se leads to eNOS dysfunction and eNOS uncoupling via increased adverse phosphorylation and *S*-glutathionylation of the enzyme (Figure 3B) [16]. We also established that *GPx-1* deficiency resulted in a phenotype of endothelial and vascular dysfunction, which was substantially potentiated by the aging process (Figure 3A). By using oxidative stress-prone *GPx-1^−/−^* mice (a model representing decreased break-down of cellular hydrogen peroxide) in a study of the aging process, we can provide a strong mechanistic link between oxidative stress, eNOS dysfunction and vascular dysfunction in aging animals (Figure 3). Most importantly, ^•^NO bioavailability was also significantly decreased in aged *GPx-1^−/−^* mice (Figure 4) supporting a dysregulation of eNOS and/or increased oxidative degradation of ^•^NO during the aging process in general and in animals with decreased antioxidant defense in particular.

As a proof of endothelial and vascular dysfunction, we showed that both, endothelium-dependent and -independent relaxation was impaired in aged *GPx-1^−/−^* mice [16]. Altered eNOS function by inactivating or uncoupling phosphorylation, PKC-dependent at Thr495 [99,100] or PYK-2-dependent at Tyr657 [101], and *S*-glutathionylation [74] leading to diminished ^•^NO bioavailability are plausible explanations for this phenotype. The deregulatory modifications of eNOS were also translated to increased uncoupling of the enzyme as envisaged by endothelial superoxide formation, which increased with age, was more pronounced in the *GPx-1^−/−^* group and nicely correlated with *S*-glutathionylation as a marker of uncoupled eNOS (Figure 3B) [16]. Since the ^•^NO target enzyme soluble guanylyl cyclase (sGC) is also subject to oxidative inactivation (*S*-oxidation, *S*-nitrosation, heme-oxidation) [102,103,104,105,106,107,108], it might be speculated that the aging process will lead to an inactivation or at least desensitization of the enzyme. At least decreased expression of sGC subunits have been reported in tissues of old animals [109,110,111]. Future studies with sGC activators will prove whether apo-sGC or heme-oxidized sGC play a role for aging-induced vascular dysfunction.

In cultured endothelial cells we demonstrated that *GPx-1* silencing increased adhesion of leukocytes, which may contribute to the observed endothelial/vascular dysfunction (e.g., by increased oxidative breakdown of ^•^NO and/or impairment of the ^•^NO-cGMP signaling cascade by infiltrated leukocytes) [16]. Furthermore, we observed an appreciable increase in cardiovascular oxidative stress and mild vascular remodeling, as detected by Sirius red and Masson’s trichrome staining (indicative for increased fibrosis of the intima and thus a decrease in intima/media ratio) [16].

*GPx-1* deficiency has been demonstrated to increase the susceptibility of cultured endothelial cells to lipopolysaccharide (LPS) by enforcing TLR4/CD14 signaling [112]. In conductance vessels, sustained overproduction of vasodilators (e.g., ^•^NO by iNOS) may reduce the responsiveness of the vasculature to these messengers because of a desensitization of the ^•^NO/cGMP pathway [113]. Indeed, increased iNOS expression and activity has been demonstrated for selenium-deficient RAW cell macrophages [114] and selenium is the precursor for selenocysteine synthesis forming the active site of GPx-1. iNOS-derived ^•^NO formation could also provide the basis for extensive protein tyrosine nitration as reported for old mice in general and *GPx-1* deficient mice in particular [16]. In summary, the progressing phenotype of low-grade inflammation in GPx-1 deficient mice during the aging process was nicely reflected by the correlation of the marker of inflammation (CD68 staining) with the overall vascular ROS formation (dihydroethidine staining (DHE staining)) in dependence of age and antioxidant defense state of the animals (Figure 3C) [16]. Since global vascular ROS formation was nicely correlated with mitochondrial ROS formation (Figure 3D), and all other parameters were linked to global vascular ROS formation, one can assume that mtROS formation has significant impact on eNOS dysregulation/uncoupling, vascular function and low-grade inflammation during the aging process [16]. This assumption is in good accordance to the reports on mtROS-driven activation of the inflammasome and expression of proinflammatory cytokines [115,116,117,118].

We observed a moderate but consistent decline in reduced thiol groups in aged *GPx-1^−/−^* mice as compared to only a minor tendency of this decline in the aged B6 wild type mice [16]. Overall, the majority of literature supports a trend of decrease in reduced thiols during the aging process, which could affect the *S*-glutathionylation pattern and accordingly the coupling state of eNOS. Smith *et al.* showed in 2006 that the decline in endothelial GSH may contribute to a change of eNOS phosphorylation pattern (decline in P-Ser1176 and increase in P-Thr494) that was associated with a loss of vascular ^•^NO bioavailability, increased proinflammatory cytokines and impaired endothelium-dependent vasodilation [119]. Recent work by Crabtree and coworkers even described an interplay of BH4 deficiency and eNOS *S*-glutathionylation in cells with diminished GTP-cyclohydrolase-1 expression providing a functional link between these two routes of eNOS uncoupling [120] that could be of relevance for the aging process as well.

According to our own previous data, mitochondrial oxidative stress increases with age and is a strong trigger of age-related endothelial/vascular dysfunction (Figure 2A) [23,28]. Using two genetic mouse models with ablated mitochondrial aldehyde dehydrogenase (*ALDH-2^−/−^*) or mitochondrial superoxide dismutase (*MnSOD^+/−^*), both important antioxidant enzymes, we could show that increased mitochondrial oxidative stress is associated with augmented oxidative mtDNA lesions (Figure 2B). Outcome from studies in genetic animal models with increased mitochondrial ROS formation (e.g., *MnSOD*- or *Trx-2*-deficiency) strongly supports an important link between cellular aging and mitochondrial dysfunction. Of note, overexpression of mitochondria-targeted catalase enhanced protection of mitochondria from ROS-induced damage and extended life span in mice [121].

Mitochondria represent an important source of reactive oxygen species, caused by electron leakage in the respiratory chain that results in univalent reduction of oxygen into O_2_^•−^. The steady state concentration of superoxide in the mitochondrial matrix is about 5- to 10-fold higher than that in the cytosolic and nuclear spaces. These apparently high mitochondrial superoxide formation rates correlate well with the reported mitochondrial oxidative DNA lesions being 10- to 20-fold higher in mitochondrial compared to nuclear DNA [29]. Cadenas and Davies proposed that susceptibility of mtDNA to oxidative damage may be ascribed to a combination of factors besides the higher superoxide formation rate in the mitochondrial matrix: unlike nuclear DNA, mitochondrial DNA lacks protective histones and enjoys only a relatively low DNA repair activity [29]. Therefore, mitochondrial 8-oxo-deoxyguanosine (8-oxo-dG) DNA lesion could represent an interesting marker for the burden of oxidative stress during the aging process [122]. According to Sastre *et al.* “mitochondrial oxidative stress should be considered a hallmark of cellular aging” [123]. The impact of mitochondrial ROS production on longevity may involve direct signal transduction pathways that are sensitive to oxidative stress, and indirect pathways related to the accumulation of oxidative damage to mitochondrial DNA, proteins, and lipids. Of note, the majority of mtDNA encodes for proteins of the mitochondrial respiratory chain and accumulation of mtDNA lesions might contribute to further uncoupling of mitochondrial electron flow at the expense of oxygen reduction to water but instead favor the formation of superoxide [124].

**Figure 5:**
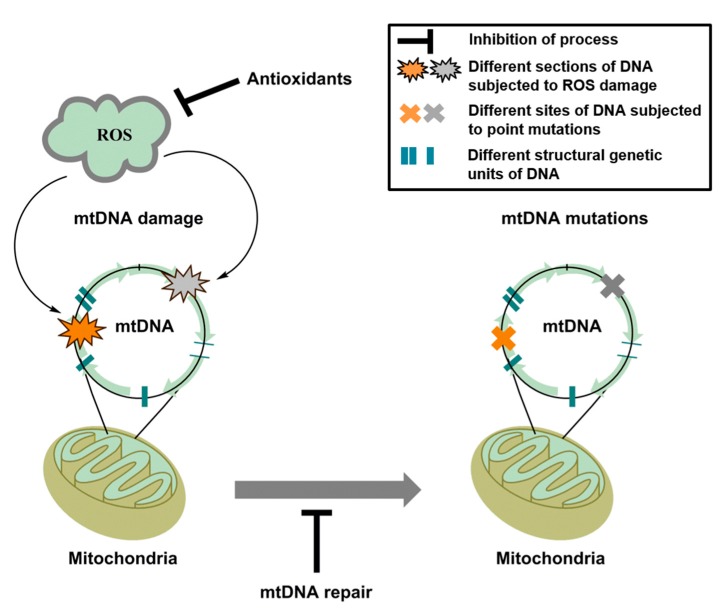
Schematic representation of mitochondrial DNA (mtDNA) damage by reactive oxygen species (ROS) leading to mtDNA mutations. Deleterious activity of ROS can be prevented by the administration of antioxidants. Damaged mtDNA can be repaired by the appropriate mtDNA repair pathway.

**Figure 6:**
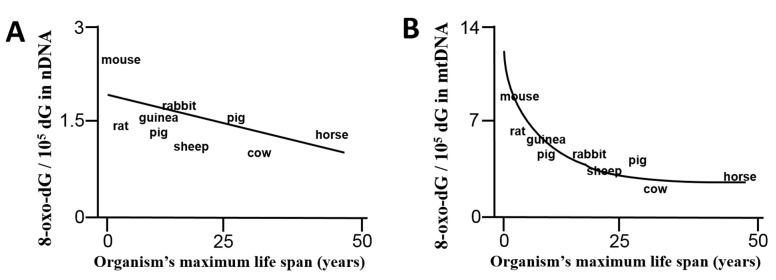
Correlation of an organism’s life span with the content of 8-oxo-dG in nuclear (**A**) or mitochondrial (**B**) DNA in cardiac tissue. Surprisingly, oxidative DNA lesions do not per se accumulate with increasing number of living years but show distinct kinetics among different animal species, tissues and probably the underlying burden of oxidative stress, efficiency of the antioxidant system and DNA repair machinery. According to Barja *et al.* [122].

The free radical hypothesis of aging highlights that reactive oxygen species are responsible for the accumulation of altered biological macromolecules such as DNA, over an organism’s lifespan [31]. Nucleic acid, in particular mitochondrial DNA (mtDNA), is regarded as a highly susceptible target for oxidant-induced mutations and deletions, which causes progressive deterioration of mitochondrial function over time (Figure 5). mtDNA deterioration belongs to a destructive cycle in which mitochondrial dysfunction further increases oxidative burden resulting in loss of cellular functions and finally apoptosis and necrosis. One of the major oxidative modifications of the mtDNA is 8-oxo-deoxyguanosine (8-oxo-dG) [125,126]. 8-oxo-dG is a mutagenic lesion and its accumulation is directly correlated with the development of pathological processes [127]. The correlation of lifespan with oxidant-induced mtDNA damage was demonstrated for the first time by Barja and co-workers [122]. These authors showed that in short-lived animals 8-oxo-dG content in nuclear and mitochondrial DNA was increased in cardiac tissue (Figure 6). These findings could be attributed to higher burden of oxidative stress in these short-lived animals (due to higher metabolic rate, less efficient antioxidant defense and/or less efficient DNA repair machinery). However, when brain tissue was investigated, accumulation of 8-oxo-dG in nuclear DNA was more pronounced in the long-lived animals (data not shown) [122]. This important study demonstrates that accumulation of oxidative DNA damage cannot per se be assumed for more living years among all different animal species, but each of them obviously has distinct kinetics of formation and repair of DNA damage, which, on top of this, depends on the specific tissue used for the quantification.

This assumption was later expanded in genetically modified mice with a proofreading-deficient mitochondrial polymerase-γ (Polγ). These mice accumulated severe mtDNA mutations, leading to mitochondrial dysfunction, increase in apoptosis and premature aging [31,128]. One of the more recent studies clearly depicted that a transgenic mouse with cardiac tissue-specific overexpression of mutated human Polγ [129] developed early aging symptoms. Elevated ROS generation and severe cardiomyopathy, typical for the “mtDNA-mutator mouse”, was also observed in these animals. Our current knowledge of maternally inherited human diseases [130], e.g., DAD-syndrome (Leu-UUR tRNA = diabetes mellitus and deafness), MELAS- (mitochondrial encephalomyopathy, lactic acidosis, and stroke-like syndrome) [131,132,133] or KSS- (Kearns-Sayre syndrome) [131,133], highlight the importance of mtDNA. Various mutations or deletions, especially in tissues with high oxygen and energy consumption such as the myocardium, increase the rate of apoptosis, free radical species formation, leading to the functional impairment of the specific organ [131,134]. Therefore, mutated mtDNA is commonly regarded as a major contributor to vascular aging and various cardiovascular disorders [131,134].

Considering that mtDNA damage accumulation with age is already widely accepted, more profound molecular explanations were provided during the last years. It has been recently shown that the observed mtDNA mutations might be mainly ascribed to errors in the replication process, to unrepaired damages, or to the spontaneous deamination of the nucleobases, indicating effects of ROS only as concomitant [135]. Investigations conducted by Itsara *et al.* [136] showed that malfunction of the replication process leads to a high accumulation of mtDNA mutations. This observation might be based on the fact that mtDNA propagates throughout the lifespan of the somatic cell, providing higher chances for the occurrence of mtDNA damage [137]. A typical mtDNA modification introduced by mitochondrial ROS is 8-hydroxy-deoxyguanosine (8-oxo-dG) [125,126]. This type of damage results in G:C to T:A transversions after DNA replication [138]. Nonetheless, this concept has not been confirmed by experimental studies, detecting only 10% of all mutations to be G:C to T:A transversions, having no correlation with the age of the animals. The most prevailing type of detected mutation was G:C to A:T transitions, attributed to the imperfect activity of mtDNA polymerase γ.

Another study by Kennedy *et al.* [139], utilizing a more clinically relevant experimental model of aging human brain, showed that mtDNA mutations during aging consist mostly of transition mutations (G:C to A:T), as depicted by ultra-sensitive detection technique and Duplex Sequencing methodology. These mtDNA mutations showed a nice correlation with the malfunction of Polγ or deamination of cytidine resulting in uracil and adenosine deamination, yielding inosine as the major mutagenic contributors in mtDNA.

Several recent papers that were summarized in the review by Cha *et al.* [140], highlight the direct correlation of mitochondrial DNA mutations with neurodegenerative diseases, in particular Huntington’s, Parkinson’s, Alzheimer’s diseases and amyotrophic lateral sclerosis (ALS). The incidence of these neurodegenerative disease increases with higher age [32]. In the setting of Alzheimer’s diseases (AD) it has been shown that post-mortem brain tissues of AD patients have elevated levels of degraded mtDNA [141] and defective base excision repair [142]. Another group was able to show that patients suffering from AD have an increased number of cytochrome C oxidase deficient neurons [143], supporting the above mentioned concept that accumulating mtDNA damage during the aging process leads to impaired biosynthesis of the respiratory chain proteins.

## 6. Aging and Mitochondrial DNA Repair

Not only the particular damage of the mtDNA molecule itself, but also the lifetime of mtDNA lesions has profound effects on the progression and characteristics of the aging phenotype. The accumulation of mtDNA damage is not only determined by the higher mtROS formation rates but also by the activity or expression levels of mitochondrial DNA repair systems. Therefore, the following part of the review concentrates on the impact of aging on the DNA repair machinery.

Taking into account the highly oxidizing environment of the mitochondria, malfunctioning of the Polγ and peculiarity of the mtDNA replication process would ultimately lead to the accumulation of mtDNA lesions. During mtDNA replication, the lagging strand is kept in the single-stranded condition for a prolonged period of time, and thereby the chances are increased for impairment of the replication. For this reason, mitochondrial protein machinery evolutionary created a multi-layered DNA repair system that is able to resolve such complex challenges [144].

One of the most extensively studied DNA repair pathways in mitochondria is base excision repair (BER) [145]. Its enzymatic pathways are identical to the ones that take place in the nucleus, starting with the modified base identification by specific glycosylase, followed by its excision, insertion of the correct nucleotide and finishing with the DNA strand ligation [146]. The family of glycosylases involved in mitochondrial BER consists of several members: OGG1 that is able to remove the most common and the most mutagenic modification introduced to the mtDNA—8-oxo-dG [147]; UNG1, which is responsible for the removal of uracil, resulting from the spontaneous deamination of the cytosine [148]; NTH1-dichotomous enzyme, possessing glycosylase and AP lyase activities, that excises pyrimidine lesions (ThyGly, FapyG and FapyA) [149]; NEIL1 and NEIL2 that are also involved in the removal of FapyG and FapyA [150]; MYH, enzyme that detects adenine placed opposite to 8-oxo-dG [151]. After specific glycosylases have identified and extracted the damaged base, the abasic sites are resolved by APE endonuclease that prepares the DNA molecule for the Polγ mediated repair process by cleaving 3′-hydroxyl and 5′-deoxyribose-5-phosphate sites [152]. Afterwards, the newly synthesized section of DNA is ligated to the rest of the molecule by DNA ligase III.

In case that the incorrect base has not been identified by BER and the replication process actually resulted in the formation of a mismatch, the adequate mismatch repair pathway (MMR) can be activated. There has been a lot of dispute in the field about the existence of this repair mechanism, since mitochondria doesn’t possess classical players of MMR, like MSH3, MSH6 or MLH1 [153]. Though recently, with the help of specific mass spectrometry analysis, a novel MMR repair protein has been identified. Y-box binding protein (YB-1) has been shown to efficiently bind DNA containing mismatches [154]. This finding was further supported by identifying 3′ and 5′-exonuclease activity of YB-1 on single stranded DNA and weak endonuclease activity on double-stranded DNA. Depletion of this enzyme by siRNA showed increased levels of mtDNA mutagenesis, confirming the presence of a unique mitochondrial MMR pathway. In contrast to the nucleus, mitochondrion doesn’t possess a nucleotide excision repair. This limitation leads to the accumulation of unrepaired bulky pyrimidine dimers, as observed upon UV irradiation.

Enzymatic reactants of the homologous recombination (HR) or non-homologous end joining (NHEJ) that are quite abundantly involved in the DNA repair inside the nucleus have not been precisely identified in the mitochondria. So far, only products of their reactivity have been detected by super-resolution and transmission electron microscopy. Among them are the following ones: 1. mtDNA deletions, which are attributed to the exo- and endonucleases activity; 2. Holiday junctions, detected in the mtDNA of the human heart and brain [155]; 3. Formation of diverse brunched structures and multiple-way junctions. All three of them are indicative of the active homologous recombination process [156]. The identification of the novel single strand annealing enzyme Mgm101 further supports this notion [157].

Recent findings on the expression levels of DNA repair systems in the matrix of aging mitochondria are quite specific for the different DNA repair systems [158]. For example, it has been shown that incidents of the homologous recombination become more abundant with the progress of aging, being strongly correlated with linear increase in the mtDNA damage [159]. Alternatively, the specific DNA glycosylase, OGG1, has been shown to be down-regulated with the onset of aging, augmenting the deleterious effects of the oxidative stress burden in the mitochondrial environment and accelerating the process of cellular senescence [160].

## 7. Crosstalk between Mitochondrial and NADPH Oxidase-Generated Reactive Oxygen and Nitrogen Species and Impact on Endothelial Function

Primary mtROS not only cause direct oxidative damage to cellular structures but may also contribute to the activation of secondary ROS sources such as the NADPH oxidases. In a feed-forward fashion, this crosstalk initiates a vicious cycle that finally may lead to eNOS dysfunction/uncoupling and impairment of endothelial function [75]. Previously, we reported on the crosstalk between mtROS and cytosolic ROS in the model of nitroglycerin-induced nitrate tolerance that is associated with increased mitochondrial oxidative stress. Nitrate tolerance is an excellent model of vascular dysfunction and oxidative stress in general [161] and mitochondrial oxidative stress and dysfunction in particular [162,163,164]. Two distinct ROS sources could be identified: endothelial dysfunction was linked to activation of NADPH oxidases, whereas vascular dysfunction was associated with mitochondrial oxidative stress [165]. Cyclosporine A, the mitochondrial permeability transition pore inhibitor, blocked this interaction (crosstalk) between mitochondrial and NADPH-dependent ROS formation and selectively improved endothelial dysfunction, whereas nitrate tolerance was not affected. In support of this observation, *gp91phox^−/−^* and *p47phox^−/−^* mice developed tolerance under nitroglycerin treatment but no endothelial dysfunction. In contrast, endothelial dysfunction and tolerance was improved by the respiratory complex I inhibitor rotenone (preventing complex I-derived ROS formation by reverse electron transfer [166]). Likewise, administration of the K_ATP_ opener diazoxide caused a nitrate tolerance-like phenomenon in control animals, whereas tolerance phenomenon was improved by the K_ATP_ inhibitor glibenclamide.

Very similar effects of these compounds have been recently demonstrated in animal and cell culture models of angiotensin-II induced hypertension [167,168]. NADPH oxidase-driven activation of mitochondrial ROS formation via K_ATP_ channels (acting as a redox switch) and changes in the membrane potential was previously proposed [169,170] and later confirmed [167]. The proposed mechanism for mtROS-driven activation of NADPH oxidase is redox-sensitive stimulation of protein kinases such as PKC and cSrc in a calcium-dependent process [97,166,171]. Synergistic formation of mtROS and NADPH oxidase derived ROS lead to uncoupling of eNOS, nitration of prostacyclin synthase and desensitization sGC [19]. Recently, we showed that this interaction (crosstalk) plays an essential role in angiotensin-II induced hypertension, vascular oxidative stress and endothelial dysfunction (envisaged by eNOS *S*-glutathionylation) [97]. In support of this proposed mechanism, increased oxidative stress per se is able to activate NADPH oxidase in a positive feedback fashion [172].

**Figure 7:**
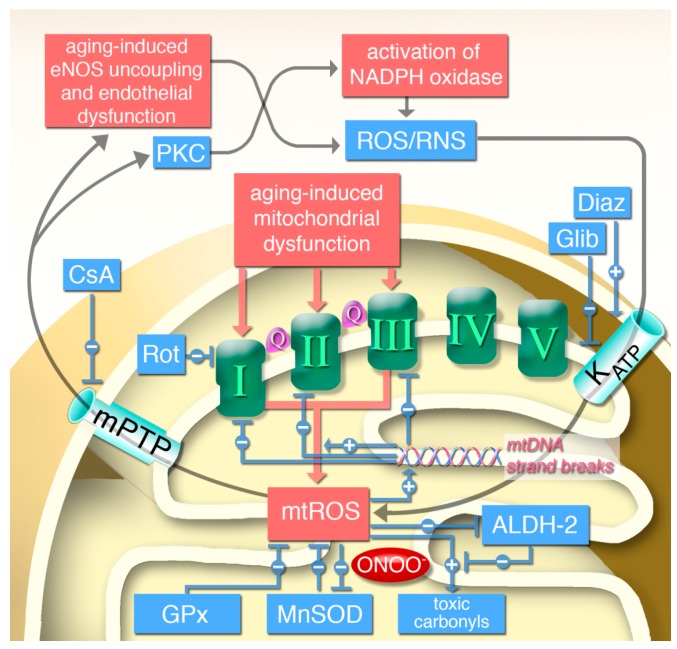
Hypothetic scheme of aging-induced vascular dysfunction and the role of mitochondria in this process. Aging-induced mitochondrial dysfunction triggers mitochondrial reactive oxygen species (mtROS) formation from respiratory complexes I, II, and III (Q = ubiquinone), whereas respiratory complexes IV and V were not reported to contribute to mtROS formation directly. Break-down of mtROS is catalyzed by glutathione peroxidase (GPx, for H_2_O_2_) or manganese superoxide dismutase (MnSOD), the latter is in turn inhibited by mitochondrial peroxynitrite (ONOO^−^) formation. mtROS increase the levels of toxic aldehydes and inhibit the mitochondrial aldehyde dehydrogenase (ALDH-2), the detoxifying enzyme of those aldehydes. Increase in mtROS and toxic aldehydes also leads to mtDNA strand breaks which leads to augmented dysfunction in respiratory chain complexes and further increase in mtROS since mtDNA encodes mainly for those respiratory complexes. mtROS also activates mitochondrial permeability transition pore (mPTP), which upon opening releases mtROS to the cytosol leading to protein kinase C (PKC)-dependent NADPH oxidase activation, eNOS uncoupling and finally to endothelial dysfunction [165]. Cytosolic reactive oxygen and nitrogen species (ROS/RNS) in turn were demonstrated to activate K_ATP_ channels, which causes alterations in mitochondrial membrane potential (C) and further augments mtROS levels [167]. Effects of rotenone (Rot), cyclosporine A (CsA), diazoxide (Diaz) and glibenclamide (Glib) have been recently demonstrated in related models of vascular dysfunction and oxidative stress, nitroglycerin-induced nitrate tolerance and angiotensin-II triggered hypertension [165,167]. + means promotion of pathways; − means inhibition of pathways. Adopted from Wenzel *et al.*, Cardiovasc. Res*.* 2008 [23]. With permission of the European Society of Cardiology. All rights reserved. © The Author and Oxford University Press 2008.

We and others propose a similar crosstalk between mitochondrial and NADPH oxidase-dependent ROS formation in the aging vasculature leading to age-related vascular dysfunction (Figure 7) [23,28,97,173]. This proposal is based on the finding that mtROS formation increases with age (and is higher in *MnSOD^+/−^* mice) and endothelial function is impaired with age (to a higher extent in *MnSOD^+/−^* mice) [23]. Of note, we and others also showed that mtROS participate in the activation of immune cells via stimulation of the phagocytic NADPH oxidase (Nox2) [97,174]. mtROS have also been reported to participate in the activation of the inflammasome and trigger the expression of proinflammatory cytokines [115,116,117,118]. In summary these data support a proinflammatory role of mtROS for progression of low-grade inflammation in the aging vasculature.

## 8. Emerging Concepts of Aging

Moosmann and coworkers have published an interesting concept, based on the observation that intramembrane accumulation of methionine exhibits antioxidant and cytoprotective properties in living cells [175]. Their findings highlight methionine as an evolutionarily selected antioxidant of the respiratory chain complexes. On top of that they provide evidence that methionine is able to redox-cycle between oxidized and reduced forms, and in such a way becomes a vital member of the antioxidant defense system. Knockout mice not expressing methionine sulfoxide reductase enzyme are characterized by a decreased lifespan and several other pathologies [176,177]. Methionine sulfoxide reductases are expressed in endothelial cells [178], are potentially involved in the prevention of endothelial dysfunction via regulation of RUNX2 transcription factor activity and biological function in endothelial cells [179]. The functional polymorphism (rs10903323 G/A) in methionine sulfoxide reductase A shows an association with the increased risk of coronary artery disease in Chinese population [180]. Moreover, vascular smooth muscle cells were protected against oxidative damage by cytosolic overexpression of methionine sulfoxide reductase A [181]. A meta-examination of 248 animal species genome sequences with known maximum lifespan, including mammals, birds, fish, insects, and helminths demonstrated that the frequency of cysteine encoded by mitochondrial DNA is a specific marker of aerobic longevity [182]: long-lived organisms synthesize respiratory chain complexes with low abundance of cysteine. These results provide distinct (indirect) support for the free radical theory of aging.

Another explanation for worsening of the physical condition with age is based on a profound role of the mitochondrial enzyme p66Shc as an adaptor protein which is implicated in mitochondrial reactive oxygen species generation and translation of oxidative signals into apoptosis [183]. Mice deficient in *p66Shc^−/−^* gene produce decreased quantities of intracellular oxidants and display 30% longer life span. In order to elucidate the function of p66Shc and its possible implication in age-associated cardiovascular diseases, a series of studies were initiated. Extensive research revealed that *p66Shc^−/−^* mice are protected from age-dependent endothelial dysfunction [184] as well as age-related risk factors such as diabetes and hypercholesterolemia. The review of Camici *et al.* focused on deciphering the novel role of the p66Shc adaptor protein and its involvement in the age-associated cardiovascular disease and pathophysiology of aging. One of the major findings of these authors is that p66Shc N terminus is activated through reversible tetramerization by forming two disulfide bonds, as a result of which it forms a redox module responsible for apoptosis initiation that is tightly associated with senescence [185]. Other systems that are involved in p66Shc regulation are two antioxidant enzymatic classes—glutathione and thioredoxin enzymes that can inactivate p66Shc through a reduction mechanism. Consequently, this forms a thiol-based redox sensor system and initiates apoptosis once cellular protection systems cannot ameliorate cellular stress anymore. Protein kinase C β and prolyl isomerase 1 effectively regulates the mitochondrial effects of the longevity-associated stimulator-p66Shc [186]. Recently, highly specialized signaling pathways leading to mitochondrial import of p66Shc that are responsible for its proapoptotic activity upon oxidative stress were analyzed [187]. In contrast, advantages for diabetic cardiovascular complications [188] and age-associated endothelial ^•^NO formation [189] were reported for genetic deletion of *p66Shc*. Last, but not least, it was validated that a p53-p66Shc signaling pathway controls intracellular redox status and contributes to increased levels of oxidative DNA damage [190] and mitochondrial oxidative stress [191]. The important role of p66Shc for vascular function is supported by recent findings that p66Shc expression correlates with the prognosis of stroke patients and post-ischemic inhibition of p66Shc reduces ischemia/reperfusion brain injury [192].

Another emerging concept in the field of aging is based on the contribution of epigenetic pathways. Epigenetic mechanisms that are involved in the aging process include alterations of the DNA methylation status, modifications of the histone tails (mainly acetylation and methylation) and changes in the expression of non-coding RNAs [193]. With increasing age, global DNA demethylation processes have been detected, leading to a DNA hypomethylation condition. This has been mostly observed on the CpG islands, which constitute regions of the DNA consisting for more than 50% of cytosine and guanine repeats [194]. On the other hand, there is a lot of information regarding sequestered, locus specific DNA hypermethylation. These nuclear regions are called senescence-associated heterochromatin foci (SAHF) [195]. The presence of such DNA segments is of high interest since they are at variance with the generally accepted fact that aging is associated with a more relaxed state of chromatin, called euchromatin [196].

One of the major epigenetic hallmarks of aging is the increased acetylation level of the histone tails. Such state is mostly attributed to the decreased level of histone deacetylases, in particular NAD^+^-dependent sirtuins [197]. The most disputed histone modification that is associated with aging is the methylation status of lysine terminal ends. Recent data support a trend of increasing tri- and di-methylation of lysine 4 and lysine 36 on the histone variant 3 (H3K4me2/3 and H3K36me3), which are considered to be gene activating marks, during the aging process. Likewise, a trend of decreasing methylation levels of lysine 27 and lysine 9 on the histone variant 3 (H3K27me3, H3K9me2/3), which are known to be gene repressing marks, was reported for the aging process. These observations support a relaxed chromatin state, which is transcriptionally active [196]. Such epigenetic and genetic alterations of the coding nucleic acid molecules might at least partially explain the increased inflammation levels due to the transcriptional gene activation that are associated with the aging process.

The field of non-coding RNAs, especially in the context of aging is currently one of the least discovered. The class of non-coding RNAs consists of microRNA, long non-coding RNAs and piwiRNA. The role of non-coding RNAs in the senescence mechanisms has been much appreciated in the form of biomarkers [198]. The novel microRNA—miR-34a, was documented as an aging marker in several tissues. Boon and colleagues have shown that miR-34a is induced in a cardiac aging model, particularly in cardiac tissue. miR-34a involvement has been also shown in acute myocardial infarction (MI). miR-34a inhibition reduces cell death and fibrosis associated with post MI conditions [199]. Investigations of Boon *et al.* clearly highlighted miR-34a and its target PNUTS as a key molecule regulating heart contractile function during the aging process by inducing DNA damage responses and telomere shortening.

Finally, the aging process is closely linked to immunosenescence and low-grade inflammation contributing to higher prevalence of metabolic syndrome, diabetes and associated cardiovascular complications [200]. Since the risk factors for cardiovascular diseases and diabetes show a clear overlap, especially at the level of inflammation, the latter could represent a key player for aging-associated disorders and increased comorbidity in the elderly [11,12,13]. Therefore, targeting of the chronic inflammatory phenotype in the elderly could represent a promising strategy to normalize the increased prevalence of comorbidities in the elderly and increase their healthspan [201]. mtROS formation is augmented with increasing age [28] and mtROS can lead to the activation of immune cells and their phagocytic NADPH oxidase, induce the inflammasome and trigger cytokine release [97,115,116,117,118,174]. Therefore, targeting mitochondrial oxidative stress in the elderly could represent another strategy to suppress the chronic inflammatory phenotype and adverse effects of the aging process. This was recently shown in an animal model for metformin-dependent AMP-activated protein kinase activation that suppressed oxidative damage and chronic inflammation in old animals and increased their lifespan and healthspan [202].

## 9. Clinical Impact

The link between endothelial dysfunction, oxidative stress and aging has not only been defined in animal models. Hypertension, dyslipidemia and atherosclerosis are precursors of cardiovascular events, like stroke or myocardial infarction [203,204]. Oxidative stress and reduced ^•^NO bioavailability impair vascular protective function of the endothelium [205]. In patients, non-invasive flow-mediated dilation (FMD) of the forearm brachial artery or plethysmography (acetylcholine-dependent vasodilation) are used to determine endothelial function *in vivo* [24] and are a predictor for cardiovascular events [3]. In patients with well-defined risk factors such as dyslipidemia or smoking, a significantly impaired acetylcholine-triggered endothelium-dependent vasodilation was observed and both risk factors are known to be associated with oxidative stress. Endothelial dysfunction of coronary arteries is directly associated with an increased risk of myocardial infarction [206]. Furthermore, Heitzer *et al.* demonstrated that patients who showed improved endothelial function after vitamin C infusion had a worse prognosis for cardiovascular events as compared to patients with low vitamin C effects (Figure 1C) [25]. These findings indicate that oxidative stress is not only a key player in the pathogenesis of endothelial dysfunction *in vivo*, but furthermore also reflects the prognosis of patients with established coronary artery disease. The concept of an increased burden of oxidative stress is broadly accepted, but nevertheless results from clinical trials investigating antioxidant vitamins like B, C, E and folic acid are disappointing (for review see [207]) and for vitamin E even increased mortality and numbers of heart failure were found (HOPE and HOPE-TOO). These findings prove what Harman already postulated in 1975 with his modified “free radical theory of aging” – that site-specific formation of ROS (e.g., in mitochondria) might be the key to understand the obvious discrepancy between association of most cardiovascular disease with oxidative stress but failure of antioxidant therapy so far. The reasons for the failure of large clinical trials on antioxidant therapy to display profound beneficial effects could be, among others, that this secondary prevention is applied to patients with already irreversible tissue/organ damage. In addition, secondary antioxidant prevention does not specifically target the defective defense and repair mechanisms (e.g., it could interfere with important intrinsic protective mechanisms such as ischemic preconditioning). Finally, the concentrations of the antioxidants reached at the sites of ROS formation could be too low (e.g., compliance was not controlled by measurement of plasma levels of the administrated antioxidants) [208]. The controversial and even contradictory results from antioxidant clinical trials or experiments manipulating antioxidants by pharmacological (or genetic approaches) suggest that aging is a complex and multifaceted process that cannot be explained by a single theory.

In humans, the incidence of hypertension, diabetes and atherosclerosis correlates with age (Figure 1A) [26,27]. In parallel, endothelial dysfunction manifests with age (Figure 1B) [24], together with oxidative stress from mitochondria and other enzymatic sources [209,210]. Aging is an independent risk factor for cardiovascular disease, which is mainly caused by endothelial dysfunction due to oxidative stress and low-grade inflammation [24,211]. Recent data provided evidence for changes in high-density lipoprotein composition and function in dependence of age [212]. Therefore, the quality of high density lipoprotein (HDL) may be another future target for therapeutic intervention in and/or diagnosis of age-related cardiovascular disease [213] because HDL quality decreases with age and this will negatively influence the endothelial function. Recently, the nitrovasodilator pentaerithrityl tetranitrate was demonstrated to provide heritable blood pressure lowering effects in hypertensive rats associated with enhanced H3K27ac and H3K4me3 and transcriptional activation of cardioprotective genes such as *eNOS*, *SOD2*, *GPx-1*, and *HO-1* [214]. Drugs like pentaerithrityl tetranitrate could represent the prototype of future epigenetic drugs that could also improve the healthspan in general and cardiovascular aging in particular. Mitochondria-targeted antioxidants could represent another promising therapeutic strategy to combat the “side effects” of the aging process. Mito-quinone improved age-related endothelial dysfunction in mice [215]. Since aging cannot be stopped like smoking [216] and unspecific antioxidant treatment using vitamins is not effective, aging-induced mitochondrial ROS formation may become a target for diagnosis and treatment of cardiovascular disease in the elderly.

## 10. Perspective

In the present review, we have provided strong evidence from our own and others studies that mitochondrial oxidative stress plays a key determinant for aging-induced impairment of cellular signaling and as a consequence cell death. There is a large body of evidence for the association of cellular aging with mitochondrial dysfunction based on genetic animal models with increased mitochondrial reactive oxygen species (ROS) formation (e.g., *MnSOD^+/−^* or *Trx-2*-deficiency). Interestingly, overexpression of catalase, another enzyme crucially involved in the antioxidant defense, enhanced protection of mitochondria from ROS, led to an extended life span in mice [121]. The increase in life span was much more pronounced when catalase was targeted to mitochondria as compared to overexpression in peroxisomes or in the nucleus. There is also evidence for an interaction (crosstalk) between mitochondrial oxidative stress and cytosolic sources of oxidative stress providing a direct link between aging and vascular dysfunction. Therefore, a better understanding of the role of mitochondria in the aging process may lead to specifically designed therapies to interfere with mitochondrial dysfunction and to delay the aging process for longevity. Since regulation of the vascular tone largely depends on a redox-balance in favor of a more reductive milieu, increased oxidative stress impairs vascular function and leads to endothelial dysfunction, atherosclerosis and other cardiovascular complications. Therefore, therapeutic intervention at the level of mitochondrial dysfunction would not only beneficially influence the aging process but also most kinds of cardiovascular diseases. The hyperacetylation state of the histone tails and impaired DNA repair capacity in aged tissues imply a therapeutic modulation of the aging process by epigenetic drugs and improved DNA repair. Since cardiovascular diseases are the main reason for mortality in the Western world and their prevalence increases with age, development of therapeutic interventions not only promises a prolonged healthspan (maybe even increased lifespan) for a large part of the world population but also represents an appreciable pharmaceutical market.

## References

[1] Kelly D.T. (1997). Paul dudley white international lecture. Our future society. A global challenge. Circulation.

[2] Lakatta E.G., Levy D. (2003). Arterial and cardiac aging: Major shareholders in cardiovascular disease enterprises: Part I: Aging arteries: A “set up” for vascular disease. Circulation.

[3] Ras R.T., Streppel M.T., Draijer R., Zock P.L. (2013). Flow-mediated dilation and cardiovascular risk prediction: A systematic review with meta-analysis. Int. J. Cardiol..

[4] Herrera M.D., Mingorance C., Rodriguez-Rodriguez R., Alvarez de Sotomayor M. (2010). Endothelial dysfunction and aging: An update. Ageing Res. Rev..

[5] Bischoff B., Silber S., Richartz B.M., Pieper L., Klotsche J., Wittchen H.U. (2006). Inadequate medical treatment of patients with coronary artery disease by primary care physicians in germany. Clin. Res. Cardiol..

[6] Burnett A.L. (2006). The role of nitric oxide in erectile dysfunction: Implications for medical therapy. J. Clin. Hypertens..

[7] Csiszar A., Toth J., Peti-Peterdi J., Ungvari Z. (2007). The aging kidney: Role of endothelial oxidative stress and inflammation. Acta Physiol. Hung..

[8] Price J.M., Hellermann A., Hellermann G., Sutton E.T. (2004). Aging enhances vascular dysfunction induced by the alzheimer’s peptide β-amyloid. Neurol. Res..

[9] Coleman H.R., Chan C.C., Ferris F.L., Chew E.Y. (2008). Age-related macular degeneration. Lancet.

[10] El Assar M., Angulo J., Rodriguez-Manas L. (2013). Oxidative stress and vascular inflammation in aging. Free Radic. Biol. Med..

[11] Cesari M., Onder G., Russo A., Zamboni V., Barillaro C., Ferrucci L., Pahor M., Bernabei R., Landi F. (2006). Comorbidity and physical function: Results from the aging and longevity study in the Sirente geographic area (iLSIRENTE study). Gerontology.

[12] Yancik R., Ershler W., Satariano W., Hazzard W., Cohen H.J., Ferrucci L. (2007). Report of the national institute on aging task force on comorbidity. J. Gerontol. Ser. A.

[13] Wieland G.D. (2005). From bedside to bench: Research in comorbidity and aging. Sci. Aging Knowl. Environ..

[14] Munzel T., Daiber A., Mulsch A. (2005). Explaining the phenomenon of nitrate tolerance. Circ. Res..

[15] Munzel T., Daiber A., Ullrich V., Mulsch A. (2005). Vascular consequences of endothelial nitric oxide synthase uncoupling for the activity and expression of the soluble guanylyl cyclase and the cGMP-dependent protein kinase. Arterioscler. Thromb. Vasc. Biol..

[16] Oelze M., Kroller-Schon S., Steven S., Lubos E., Doppler C., Hausding M., Tobias S., Brochhausen C., Li H., Torzewski M. (2014). Glutathione peroxidase-1 deficiency potentiates dysregulatory modifications of endothelial nitric oxide synthase and vascular dysfunction in aging. Hypertension.

[17] Van der Loo B., Labugger R., Skepper J.N., Bachschmid M., Kilo J., Powell J.M., Palacios-Callender M., Erusalimsky J.D., Quaschning T., Malinski T. (2000). Enhanced peroxynitrite formation is associated with vascular aging. J. Exp. Med..

[18] Forstermann U., Sessa W.C. (2012). Nitric oxide synthases: Regulation and function. Eur. Heart J..

[19] Daiber A., Oelze M., Daub S., Steven S., Schuff A., Kroller-Schon S., Hausding M., Wenzel P., Schulz E., Gori T., Laher I. (2014). Vascular redox signaling, redox switches in endothelial nitric oxide synthase and endothelial dysfunction. Systems Biology of Free Radicals and Antioxidants.

[20] Donato A.J., Gano L.B., Eskurza I., Silver A.E., Gates P.E., Jablonski K., Seals D.R. (2009). Vascular endothelial dysfunction with aging: Endothelin-1 and endothelial nitric oxide synthase. Am. J. Phys. Heart Circ. Physiol..

[21] Donato A.J., Magerko K.A., Lawson B.R., Durrant J.R., Lesniewski L.A., Seals D.R. (2011). SIRT-1 and vascular endothelial dysfunction with ageing in mice and humans. J. Physiol..

[22] Higashi Y., Sasaki S., Nakagawa K., Kimura M., Noma K., Hara K., Jitsuiki D., Goto C., Oshima T., Chayama K. (2006). Tetrahydrobiopterin improves aging-related impairment of endothelium-dependent vasodilation through increase in nitric oxide production. Atherosclerosis.

[23] Wenzel P., Schuhmacher S., Kienhofer J., Muller J., Hortmann M., Oelze M., Schulz E., Treiber N., Kawamoto T., Scharffetter-Kochanek K. (2008). Manganese superoxide dismutase and aldehyde dehydrogenase deficiency increase mitochondrial oxidative stress and aggravate age-dependent vascular dysfunction. Cardiovasc. Res..

[24] Gerhard M., Roddy M.A., Creager S.J., Creager M.A. (1996). Aging progressively impairs endothelium-dependent vasodilation in forearm resistance vessels of humans. Hypertension.

[25] Heitzer T., Schlinzig T., Krohn K., Meinertz T., Munzel T. (2001). Endothelial dysfunction, oxidative stress, and risk of cardiovascular events in patients with coronary artery disease. Circulation.

[26] Savji N., Rockman C.B., Skolnick A.H., Guo Y., Adelman M.A., Riles T., Berger J.S. (2013). Association between advanced age and vascular disease in different arterial territories: A population database of over 3.6 million subjects. J. Am. Coll. Cardiol..

[27] Ong K.L., Cheung B.M., Man Y.B., Lau C.P., Lam K.S. (2007). Prevalence, awareness, treatment, and control of hypertension among united states adults 1999–2004. Hypertension.

[28] Daiber A., Kienhoefer J., Zee R., Ullrich V., van der Loo B., Bachschmid M., Bondy S.C., Maiese K. (2010). The role of mitochondrial reactive oxygen species formation for age-induced vascular dysfunction. Aging and Age-Related Disorders.

[29] Cadenas E., Davies K.J. (2000). Mitochondrial free radical generation, oxidative stress, and aging. Free Radic. Biol. Med..

[30] Lenaz G., Bovina C., D’Aurelio M., Fato R., Formiggini G., Genova M.L., Giuliano G., Pich M.M., Paolucci U., Castelli G.P. (2002). Role of mitochondria in oxidative stress and aging. Ann. N. Y. Acad. Sci..

[31] Kujoth G.C., Hiona A., Pugh T.D., Someya S., Panzer K., Wohlgemuth S.E., Hofer T., Seo A.Y., Sullivan R., Jobling W.A. (2005). Mitochondrial DNA mutations, oxidative stress, and apoptosis in mammalian aging. Science.

[32] Yin F., Boveris A., Cadenas E. (2014). Mitochondrial energy metabolism and redox signaling in brain aging and neurodegeneration. Antioxid. Redox Signal..

[33] Cheng Z.J., Vapaatalo H., Mervaala E. (2005). Angiotensin II and vascular inflammation. Med. Sci. Monit..

[34] Lau D., Baldus S. (2006). Myeloperoxidase and its contributory role in inflammatory vascular disease. Pharmacol. Ther..

[35] Willerson J.T., Golino P., Eidt J., Campbell W.B., Buja L.M. (1989). Specific platelet mediators and unstable coronary artery lesions. Experimental evidence and potential clinical implications. Circulation.

[36] Seals D.R., Jablonski K.L., Donato A.J. (2011). Aging and vascular endothelial function in humans. Clin. Sci..

[37] Tanaka H., Dinenno F.A., Seals D.R. (2000). Age-related increase in femoral intima-media thickness in healthy humans. Arterioscler. Thromb. Vasc. Biol..

[38] Crimi E., Ignarro L.J., Napoli C. (2007). Microcirculation and oxidative stress. Free Radic. Res..

[39] Mayhan W.G., Arrick D.M., Sharpe G.M., Sun H. (2008). Age-related alterations in reactivity of cerebral arterioles: Role of oxidative stress. Microcirculation.

[40] Militante J., Lombardini J.B. (2004). Age-related retinal degeneration in animal models of aging: Possible involvement of taurine deficiency and oxidative stress. Neurochem. Res..

[41] Fischer R., Maier O. (2015). Interrelation of oxidative stress and inflammation in neurodegenerative disease: Role of TNF. Oxidative Med. Cell. Longev..

[42] Blasiak J., Petrovski G., Vereb Z., Facsko A., Kaarniranta K. (2014). Oxidative stress, hypoxia, and autophagy in the neovascular processes of age-related macular degeneration. BioMed. Res. Int..

[43] Harman D. (1956). Aging: A theory based on free radical and radiation chemistry. J. Gerontol..

[44] Waters W.A. (1946). Some recent developments in the chemistry of free radicals. J. Chem. Soc..

[45] Rogell B., Dean R., Lemos B., Dowling D.K. (2014). Mito-nuclear interactions as drivers of gene movement on and off the X-chromosome. BMC Genomics.

[46] Thomas D.D., Ridnour L.A., Isenberg J.S., Flores-Santana W., Switzer C.H., Donzelli S., Hussain P., Vecoli C., Paolocci N., Ambs S. (2008). The chemical biology of nitric oxide: Implications in cellular signaling. Free Radic. Biol. Med..

[47] Van der Loo B., Bachschmid M., Skepper J.N., Labugger R., Schildknecht S., Hahn R., Mussig E., Gygi D., Luscher T.F. (2006). Age-associated cellular relocation of Sod 1 as a self-defense is a futile mechanism to prevent vascular aging. Biochem. Biophys. Res. Commun..

[48] De Haan J.B., Bladier C., Lotfi-Miri M., Taylor J., Hutchinson P., Crack P.J., Hertzog P., Kola I. (2004). Fibroblasts derived from Gpx1 knockout mice display senescent-like features and are susceptible to H_2_O_2_-mediated cell death. Free Radic. Biol. Med..

[49] Altschmied J., Haendeler J. (2009). Thioredoxin-1 and endothelial cell aging: Role in cardiovascular diseases. Antioxid. Redox Signal..

[50] Go Y.M., Jones D.P. (2010). Redox control systems in the nucleus: Mechanisms and functions. Antioxid. Redox Signal..

[51] Salmon A.B., Richardson A., Perez V.I. (2010). Update on the oxidative stress theory of aging: Does oxidative stress play a role in aging or healthy aging?. Free Radic. Biol. Med..

[52] Brown K.A., Didion S.P., Andresen J.J., Faraci F.M. (2007). Effect of aging, MnSOD deficiency, and genetic background on endothelial function: Evidence for MnSOD haploinsufficiency. Arterioscler. Thromb. Vasc. Biol..

[53] Didion S.P., Kinzenbaw D.A., Schrader L.I., Faraci F.M. (2006). Heterozygous CuZn superoxide dismutase deficiency produces a vascular phenotype with aging. Hypertension.

[54] Goldstein S., Czapski G., Lind J., Merenyi G. (2000). Tyrosine nitration by simultaneous generation of ^•^NO and O^•^_2_ under physiological conditions. How the radicals do the job. J. Biol. Chem..

[55] Perez V.I., Bokov A., van Remmen H., Mele J., Ran Q., Ikeno Y., Richardson A. (2009). Is the oxidative stress theory of aging dead?. Biochim. Biophys. Acta.

[56] Muller F.L., Lustgarten M.S., Jang Y., Richardson A., van Remmen H. (2007). Trends in oxidative aging theories. Free Radic. Biol. Med..

[57] Lebovitz R.M., Zhang H., Vogel H., Cartwright J., Dionne L., Lu N., Huang S., Matzuk M.M. (1996). Neurodegeneration, myocardial injury, and perinatal death in mitochondrial superoxide dismutase-deficient mice. Proc. Natl. Acad. Sci. USA.

[58] Li Y., Huang T.T., Carlson E.J., Melov S., Ursell P.C., Olson J.L., Noble L.J., Yoshimura M.P., Berger C., Chan P.H. (1995). Dilated cardiomyopathy and neonatal lethality in mutant mice lacking manganese superoxide dismutase. Nat. Genet..

[59] Jang Y.C., Remmen H.V. (2009). The mitochondrial theory of aging: Insight from transgenic and knockout mouse models. Exp. Gerontol..

[60] Dai D.F., Chiao Y.A., Marcinek D.J., Szeto H.H., Rabinovitch P.S. (2014). Mitochondrial oxidative stress in aging and healthspan. Longev. Healthspan.

[61] Hamilton R.T., Walsh M.E., van Remmen H. (2012). Mouse models of oxidative stress indicate a role for modulating healthy aging. J. Clin. Exp. Pathol..

[62] Berry A., Cirulli F. (2013). The *p66Shc* gene paves the way for healthspan: Evolutionary and mechanistic perspectives. Neurosci. Biobehav. Rev..

[63] Wanagat J., Dai D.F., Rabinovitch P. (2010). Mitochondrial oxidative stress and mammalian healthspan. Mech. Ageing Dev..

[64] Sies H. (2015). Oxidative stress: A concept in redox biology and medicine. Redox Biol..

[65] Beckman J.S., Koppenol W.H. (1996). Nitric oxide, superoxide, and peroxynitrite: The good, the bad, and ugly. Am. J. Physiol..

[66] Daiber A., Bachschmid M. (2007). Enzyme inhibition by peroxynitrite-mediated tyrosine nitration and thiol oxidation. Curr. Enzym. Inhib..

[67] Beckman J.S. (2002). Protein tyrosine nitration and peroxynitrite. FASEB J..

[68] Quijano C., Alvarez B., Gatti R.M., Augusto O., Radi R. (1997). Pathways of peroxynitrite oxidation of thiol groups. Biochem. J..

[69] MacMillan-Crow L.A., Crow J.P., Kerby J.D., Beckman J.S., Thompson J.A. (1996). Nitration and inactivation of manganese superoxide dismutase in chronic rejection of human renal allografts. Proc. Natl. Acad. Sci. USA.

[70] Kuzkaya N., Weissmann N., Harrison D.G., Dikalov S. (2003). Interactions of peroxynitrite, tetrahydrobiopterin, ascorbic acid, and thiols: Implications for uncoupling endothelial nitric-oxide synthase. J. Biol. Chem..

[71] Schulz E., Jansen T., Wenzel P., Daiber A., Munzel T. (2008). Nitric oxide, tetrahydrobiopterin, oxidative stress, and endothelial dysfunction in hypertension. Antioxid. Redox Signal..

[72] Yoshida Y.I., Eda S., Masada M. (2000). Alterations of tetrahydrobiopterin biosynthesis and pteridine levels in mouse tissues during growth and aging. Brain Dev..

[73] Blackwell K.A., Sorenson J.P., Richardson D.M., Smith L.A., Suda O., Nath K., Katusic Z.S. (2004). Mechanisms of aging-induced impairment of endothelium-dependent relaxation: Role of tetrahydrobiopterin. Am. J. Physiol. Heart Circ. Physiol..

[74] Chen C.A., Wang T.Y., Varadharaj S., Reyes L.A., Hemann C., Talukder M.A., Chen Y.R., Druhan L.J., Zweier J.L. (2010). *S*-glutathionylation uncouples eNOS and regulates its cellular and vascular function. Nature.

[75] Schulz E., Wenzel P., Munzel T., Daiber A. (2014). Mitochondrial redox signaling: Interaction of mitochondrial reactive oxygen species with other sources of oxidative stress. Antioxid. Redox Signal..

[76] Zou M.H., Shi C., Cohen R.A. (2002). Oxidation of the zinc-thiolate complex and uncoupling of endothelial nitric oxide synthase by peroxynitrite. J. Clin. Investig..

[77] Forstermann U., Munzel T. (2006). Endothelial nitric oxide synthase in vascular disease: From marvel to menace. Circulation.

[78] Soucy K.G., Ryoo S., Benjo A., Lim H.K., Gupta G., Sohi J.S., Elser J., Aon M.A., Nyhan D., Shoukas A.A. (2006). Impaired shear stress-induced nitric oxide production through decreased NOS phosphorylation contributes to age-related vascular stiffness. J. Appl. Physiol..

[79] Cave A.C., Brewer A.C., Narayanapanicker A., Ray R., Grieve D.J., Walker S., Shah A.M. (2006). Nadph oxidases in cardiovascular health and disease. Antioxid. Redox signal..

[80] Griendling K.K., Sorescu D., Ushio-Fukai M. (2000). NAD(P)H oxidase: Role in cardiovascular biology and disease. Circ. Res..

[81] Paneni F., Osto E., Costantino S., Mateescu B., Briand S., Coppolino G., Perna E., Mocharla P., Akhmedov A., Kubant R. (2013). Deletion of the activated protein-1 transcription factor JunD induces oxidative stress and accelerates age-related endothelial dysfunction. Circulation.

[82] Roubenoff R., Harris T.B., Abad L.W., Wilson P.W., Dallal G.E., Dinarello C.A. (1998). Monocyte cytokine production in an elderly population: Effect of age and inflammation. J. Gerontol. Ser. A.

[83] Moe K.T., Aulia S., Jiang F., Chua Y.L., Koh T.H., Wong M.C., Dusting G.J. (2006). Differential upregulation of Nox homologues of NADPH oxidase by tumor necrosis factor-α in human aortic smooth muscle and embryonic kidney cells. J. Cell. Mol. Med..

[84] Karbach S., Wenzel P., Waisman A., Munzel T., Daiber A. (2014). eNOS uncoupling in cardiovascular diseases—The role of oxidative stress and inflammation. Curr. Pharm. Des..

[85] Nandi J., Saud B., Zinkievich J.M., Yang Z.J., Levine R.A. (2010). TNF-α modulates INOS expression in an experimental rat model of indomethacin-induced jejunoileitis. Mol. Cell. Biochem..

[86] Ungvari Z., Csiszar A., Edwards J.G., Kaminski P.M., Wolin M.S., Kaley G., Koller A. (2003). Increased superoxide production in coronary arteries in hyperhomocysteinemia: Role of tumor necrosis factor-α, NAD(P)H oxidase, and inducible nitric oxide synthase. Arterioscler. Thromb. Vasc. Biol..

[87] Busik J.V., Mohr S., Grant M.B. (2008). Hyperglycemia-induced reactive oxygen species toxicity to endothelial cells is dependent on paracrine mediators. Diabetes.

[88] Csiszar A., Labinskyy N., Smith K., Rivera A., Orosz Z., Ungvari Z. (2007). Vasculoprotective effects of anti-tumor necrosis factor-α treatment in aging. Am. J. Pathol..

[89] Ferrucci L., Corsi A., Lauretani F., Bandinelli S., Bartali B., Taub D.D., Guralnik J.M., Longo D.L. (2005). The origins of age-related proinflammatory state. Blood.

[90] Wenzel P., Knorr M., Kossmann S., Stratmann J., Hausding M., Schuhmacher S., Karbach S.H., Schwenk M., Yogev N., Schulz E. (2011). Lysozyme M-positive monocytes mediate angiotensin II-induced arterial hypertension and vascular dysfunction. Circulation.

[91] Guzik T.J., Hoch N.E., Brown K.A., McCann L.A., Rahman A., Dikalov S., Goronzy J., Weyand C., Harrison D.G. (2007). Role of the T cell in the genesis of angiotensin II induced hypertension and vascular dysfunction. J. Exp. Med..

[92] Harman D. (1972). The biologic clock: The mitochondria?. J. Am. Geriatr. Soc..

[93] Lewis P., Stefanovic N., Pete J., Calkin A.C., Giunti S., Thallas-Bonke V., Jandeleit-Dahm K.A., Allen T.J., Kola I., Cooper M.E. (2007). Lack of the antioxidant enzyme glutathione peroxidase-1 accelerates atherosclerosis in diabetic apolipoprotein e-deficient mice. Circulation.

[94] Forgione M.A., Cap A., Liao R., Moldovan N.I., Eberhardt R.T., Lim C.C., Jones J., Goldschmidt-Clermont P.J., Loscalzo J. (2002). Heterozygous cellular glutathione peroxidase deficiency in the mouse: Abnormalities in vascular and cardiac function and structure. Circulation.

[95] Chrissobolis S., Didion S.P., Kinzenbaw D.A., Schrader L.I., Dayal S., Lentz S.R., Faraci F.M. (2008). Glutathione peroxidase-1 plays a major role in protecting against angiotensin II-induced vascular dysfunction. Hypertension.

[96] Blankenberg S., Rupprecht H.J., Bickel C., Torzewski M., Hafner G., Tiret L., Smieja M., Cambien F., Meyer J., Lackner K.J. (2003). Glutathione peroxidase 1 activity and cardiovascular events in patients with coronary artery disease. N. Engl. J. Med..

[97] Kroller-Schon S., Steven S., Kossmann S., Scholz A., Daub S., Oelze M., Xia N., Hausding M., Mikhed Y., Zinssius E. (2014). Molecular mechanisms of the crosstalk between mitochondria and NADPH oxidase through reactive oxygen species-studies in white blood cells and in animal models. Antioxid. Redox Signal..

[98] Hausding M., Jurk K., Daub S., Kroller-Schon S., Stein J., Schwenk M., Oelze M., Mikhed Y., Kerahrodi J.G., Kossmann S. (2013). CD40L contributes to angiotensin II-induced pro-thrombotic state, vascular inflammation, oxidative stress and endothelial dysfunction. Basic Res. Cardiol..

[99] Fleming I., Fisslthaler B., Dimmeler S., Kemp B.E., Busse R. (2001). Phosphorylation of Thr^495^ regulates Ca^2+^/calmodulin-dependent endothelial nitric oxide synthase activity. Circ. Res..

[100] Lin M.I., Fulton D., Babbitt R., Fleming I., Busse R., Pritchard K.A., Sessa W.C. (2003). Phosphorylation of threonine 497 in endothelial nitric-oxide synthase coordinates the coupling of l-arginine metabolism to efficient nitric oxide production. J. Biol. Chem..

[101] Loot A.E., Schreiber J.G., Fisslthaler B., Fleming I. (2009). Angiotensin ii impairs endothelial function via tyrosine phosphorylation of the endothelial nitric oxide synthase. J. Exp. Med..

[102] Brune B., Schmidt K.U., Ullrich V. (1990). Activation of soluble guanylate cyclase by carbon monoxide and inhibition by superoxide anion. Eur. J. Biochem..

[103] Weber M., Lauer N., Mulsch A., Kojda G. (2001). The effect of peroxynitrite on the catalytic activity of soluble guanylyl cyclase. Free Radic. Biol. Med..

[104] Artz J.D., Schmidt B., McCracken J.L., Marletta M.A. (2002). Effects of nitroglycerin on soluble guanylate cyclase: Implications for nitrate tolerance. J. Biol. Chem..

[105] Crassous P.A., Couloubaly S., Huang C., Zhou Z., Baskaran P., Kim D.D., Papapetropoulos A., Fioramonti X., Duran W.N., Beuve A. (2012). Soluble guanylyl cyclase is a target of angiotensin II-induced nitrosative stress in a hypertensive rat model. Am. J. Physiol. Heart Circ. Physiol..

[106] Mayer B., Kleschyov A.L., Stessel H., Russwurm M., Munzel T., Koesling D., Schmidt K. (2009). Inactivation of soluble guanylate cyclase by stoichiometric *S*-nitrosation. Mol. Pharmacol..

[107] Sayed N., Kim D.D., Fioramonti X., Iwahashi T., Duran W.N., Beuve A. (2008). Nitroglycerin-induced *S*-nitrosylation and desensitization of soluble guanylyl cyclase contribute to nitrate tolerance. Circ. Res..

[108] Stasch J.P., Schmidt P.M., Nedvetsky P.I., Nedvetskaya T.Y., H S A.K., Meurer S., Deile M., Taye A., Knorr A., Lapp H. (2006). Targeting the heme-oxidized nitric oxide receptor for selective vasodilatation of diseased blood vessels. J. Clin. Investig..

[109] Chen L., Daum G., Fischer J.W., Hawkins S., Bochaton-Piallat M.L., Gabbiani G., Clowes A.W. (2000). Loss of expression of the β subunit of soluble guanylyl cyclase prevents nitric oxide-mediated inhibition of DNA synthesis in smooth muscle cells of old rats. Circ. Res..

[110] Ruetten H., Zabel U., Linz W., Schmidt H.H. (1999). Downregulation of soluble guanylyl cyclase in young and aging spontaneously hypertensive rats. Circ. Res..

[111] Kloss S., Bouloumie A., Mulsch A. (2000). Aging and chronic hypertension decrease expression of rat aortic soluble guanylyl cyclase. Hypertension.

[112] Lubos E., Mahoney C.E., Leopold J.A., Zhang Y.Y., Loscalzo J., Handy D.E. (2010). Glutathione peroxidase-1 modulates lipopolysaccharide-induced adhesion molecule expression in endothelial cells by altering CD14 expression. FASEB J..

[113] Kessler P., Bauersachs J., Busse R., Schini-Kerth V.B. (1997). Inhibition of inducible nitric oxide synthase restores endothelium-dependent relaxations in proinflammatory mediator-induced blood vessels. Arterioscler. Thromb. Vasc. Biol..

[114] Prabhu K.S., Zamamiri-Davis F., Stewart J.B., Thompson J.T., Sordillo L.M., Reddy C.C. (2002). Selenium deficiency increases the expression of inducible nitric oxide synthase in RAW 264.7 macrophages: Role of nuclear factor-κB in up-regulation. Biochem. J..

[115] Bulua A.C., Simon A., Maddipati R., Pelletier M., Park H., Kim K.Y., Sack M.N., Kastner D.L., Siegel R.M. (2011). Mitochondrial reactive oxygen species promote production of proinflammatory cytokines and are elevated in TNFR1-associated periodic syndrome (TRAPS). J. Exp. Med..

[116] West A.P., Brodsky I.E., Rahner C., Woo D.K., Erdjument-Bromage H., Tempst P., Walsh M.C., Choi Y., Shadel G.S., Ghosh S. (2011). TLR signalling augments macrophage bactericidal activity through mitochondrial ROS. Nature.

[117] Zhou R., Yazdi A.S., Menu P., Tschopp J. (2011). A role for mitochondria in NLRP3 inflammasome activation. Nature.

[118] Zhou R., Tardivel A., Thorens B., Choi I., Tschopp J. (2010). Thioredoxin-interacting protein links oxidative stress to inflammasome activation. Nat. Immunol..

[119] Smith A.R., Visioli F., Frei B., Hagen T.M. (2006). Age-related changes in endothelial nitric oxide synthase phosphorylation and nitric oxide dependent vasodilation: Evidence for a novel mechanism involving sphingomyelinase and ceramide-activated phosphatase 2A. Aging Cell.

[120] Crabtree M.J., Brixey R., Batchelor H., Hale A.B., Channon K.M. (2013). Integrated redox sensor and effector functions for tetrahydrobiopterin- and glutathionylation-dependent endothelial nitric-oxide synthase uncoupling. J. Biol. Chem..

[121] Schriner S.E., Linford N.J., Martin G.M., Treuting P., Ogburn C.E., Emond M., Coskun P.E., Ladiges W., Wolf N., van Remmen H. (2005). Extension of murine life span by overexpression of catalase targeted to mitochondria. Science.

[122] Barja G., Herrero A. (2000). Oxidative damage to mitochondrial DNA is inversely related to maximum life span in the heart and brain of mammals. FASEB J..

[123] Sastre J., Pallardo F.V., Vina J. (2003). The role of mitochondrial oxidative stress in aging. Free Radic. Biol. Med..

[124] Madamanchi N.R., Runge M.S. (2007). Mitochondrial dysfunction in atherosclerosis. Circ. Res..

[125] De Souza-Pinto N.C., Eide L., Hogue B.A., Thybo T., Stevnsner T., Seeberg E., Klungland A., Bohr V.A. (2001). Repair of 8-oxodeoxyguanosine lesions in mitochondrial DNA depends on the oxoguanine DNA glycosylase (OGG1) gene and 8-oxoguanine accumulates in the mitochondrial dna of OGG1-defective mice. Cancer Res..

[126] De Souza-Pinto N.C., Hogue B.A., Bohr V.A. (2001). DNA repair and aging in mouse liver: 8-oxodG glycosylase activity increase in mitochondrial but not in nuclear extracts. Free Radic. Biol. Med..

[127] Souza-Pinto N.C., Croteau D.L., Hudson E.K., Hansford R.G., Bohr V.A. (1999). Age-associated increase in 8-oxo-deoxyguanosine glycosylase/ap lyase activity in rat mitochondria. Nucleic Acids Res..

[128] Trifunovic A., Wredenberg A., Falkenberg M., Spelbrink J.N., Rovio A.T., Bruder C.E., Bohlooly Y.M., Gidlof S., Oldfors A., Wibom R. (2004). Premature ageing in mice expressing defective mitochondrial DNA polymerase. Nature.

[129] Lewis W., Day B.J., Kohler J.J., Hosseini S.H., Chan S.S., Green E.C., Haase C.P., Keebaugh E.S., Long R., Ludaway T. (2007). Decreased mtDNA, oxidative stress, cardiomyopathy, and death from transgenic cardiac targeted human mutant polymerase γ. Lab. Investig..

[130] Finsterer J. (2006). Overview on visceral manifestations of mitochondrial disorders. Neth. J. Med..

[131] Anan R., Nakagawa M., Miyata M., Higuchi I., Nakao S., Suehara M., Osame M., Tanaka H. (1995). Cardiac involvement in mitochondrial diseases. A study on 17 patients with documented mitochondrial DNA defects. Circulation.

[132] Pinsky D.J., Oz M.C., Koga S., Taha Z., Broekman M.J., Marcus A.J., Liao H., Naka Y., Brett J., Cannon P.J. (1994). Cardiac preservation is enhanced in a heterotopic rat transplant model by supplementing the nitric oxide pathway. J. Clin. Investig..

[133] Zeviani M., di Donato S. (2004). Mitochondrial disorders. Brain.

[134] Ballinger S.W., Patterson C., Knight-Lozano C.A., Burow D.L., Conklin C.A., Hu Z., Reuf J., Horaist C., Lebovitz R., Hunter G.C. (2002). Mitochondrial integrity and function in atherogenesis. Circulation.

[135] Sevini F., Giuliani C., Vianello D., Giampieri E., Santoro A., Biondi F., Garagnani P., Passarino G., Luiselli D., Capri M. (2014). mtDNA mutations in human aging and longevity: Controversies and new perspectives opened by high-throughput technologies. Exp.Gerontol..

[136] Itsara L.S., Kennedy S.R., Fox E.J., Yu S., Hewitt J.J., Sanchez-Contreras M., Cardozo-Pelaez F., Pallanck L.J. (2014). Oxidative stress is not a major contributor to somatic mitochondrial DNA mutations. PLoS Genet..

[137] Larsson N.G. (2010). Somatic mitochondrial DNA mutations in mammalian aging. Annu. Rev. Biochem..

[138] De Bont R., van Larebeke N. (2004). Endogenous DNA damage in humans: A review of quantitative data. Mutagenesis.

[139] Kennedy S.R., Salk J.J., Schmitt M.W., Loeb L.A. (2013). Ultra-sensitive sequencing reveals an age-related increase in somatic mitochondrial mutations that are inconsistent with oxidative damage. PLoS Genet..

[140] Cha M.Y., Kim D.K., Mook-Jung I. (2015). The role of mitochondrial DNA mutation on neurodegenerative diseases. Exp. Mol. Med..

[141] Reddy P.H. (2009). Amyloid β, mitochondrial structural and functional dynamics in alzheimer’s disease. Exp. Neurol..

[142] Canugovi C., Shamanna R.A., Croteau D.L., Bohr V.A. (2014). Base excision DNA repair levels in mitochondrial lysates of alzheimer’s disease. Neurobiol. Aging.

[143] Krishnan K.J., Ratnaike T.E., de Gruyter H.L., Jaros E., Turnbull D.M. (2012). Mitochondrial DNA deletions cause the biochemical defect observed in alzheimer’s disease. Neurobiol. Aging.

[144] Muftuoglu M., Mori M.P., de Souza-Pinto N.C. (2014). Formation and repair of oxidative damage in the mitochondrial DNA. Mitochondrion.

[145] Liu P., Demple B. (2010). DNA repair in mammalian mitochondria: Much more than we thought?. Environ. Mol. Mutagen..

[146] Bogenhagen D.F. (1999). Repair of mtDNA in vertebrates. Am. J. Hum. Genet..

[147] Nishioka K., Ohtsubo T., Oda H., Fujiwara T., Kang D., Sugimachi K., Nakabeppu Y. (1999). Expression and differential intracellular localization of two major forms of human 8-oxoguanine DNA glycosylase encoded by alternatively spliced OGG1 mRNAs. Mol. Biol. Cell.

[148] Nilsen H., Otterlei M., Haug T., Solum K., Nagelhus T.A., Skorpen F., Krokan H.E. (1997). Nuclear and mitochondrial uracil-DNA glycosylases are generated by alternative splicing and transcription from different positions in the UNG gene. Nucleic Acids Res..

[149] Ikeda S., Kohmoto T., Tabata R., Seki Y. (2002). Differential intracellular localization of the human and mouse endonuclease III homologs and analysis of the sorting signals. DNA Repair.

[150] Hu J., de Souza-Pinto N.C., Haraguchi K., Hogue B.A., Jaruga P., Greenberg M.M., Dizdaroglu M., Bohr V.A. (2005). Repair of formamidopyrimidines in DNA involves different glycosylases: Role of the OGG1, NTH1, and NEIL1 enzymes. J. Biol. Chem..

[151] Ohtsubo T., Nishioka K., Imaiso Y., Iwai S., Shimokawa H., Oda H., Fujiwara T., Nakabeppu Y. (2000). Identification of human muty homolog (hMYH) as a repair enzyme for 2-hydroxyadenine in DNA and detection of multiple forms of hMYH located in nuclei and mitochondria. Nucleic Acids Res..

[152] Park J.S., Kim H.L., Kim Y.J., Weon J.I., Sung M.K., Chung H.W., Seo Y.R. (2014). Human AP endonuclease 1: A potential marker for the prediction of environmental carcinogenesis risk. Oxidative Med. Cell. Longev..

[153] Mason P.A., Matheson E.C., Hall A.G., Lightowlers R.N. (2003). Mismatch repair activity in mammalian mitochondria. Nucleic Acids Res..

[154] De Souza-Pinto N.C., Mason P.A., Hashiguchi K., Weissman L., Tian J., Guay D., Lebel M., Stevnsner T.V., Rasmussen L.J., Bohr V.A. (2009). Novel DNA mismatch-repair activity involving YB-1 in human mitochondria. DNA Repair.

[155] Pohjoismaki J.L., Goffart S., Tyynismaa H., Willcox S., Ide T., Kang D., Suomalainen A., Karhunen P.J., Griffith J.D., Holt I.J. (2009). Human heart mitochondrial DNA is organized in complex catenated networks containing abundant four-way junctions and replication forks. J. Biol. Chem..

[156] Chen X.J. (2013). Mechanism of homologous recombination and implications for aging-related deletions in mitochondrial DNA. Microbiol. Mol. Biol. Rev..

[157] Chen X.J., Guan M.X., Clark-Walker G.D. (1993). MGM101, a nuclear gene involved in maintenance of the mitochondrial genome in saccharomyces cerevisiae. Nucleic Acids Res..

[158] Gredilla R., Garm C., Stevnsner T. (2012). Nuclear and mitochondrial DNA repair in selected eukaryotic aging model systems. Oxidative Med. Cell. Longev..

[159] Bender A., Krishnan K.J., Morris C.M., Taylor G.A., Reeve A.K., Perry R.H., Jaros E., Hersheson J.S., Betts J., Klopstock T. (2006). High levels of mitochondrial DNA deletions in substantia nigra neurons in aging and Parkinson disease. Nat. Genet..

[160] Chen D., Cao G., Hastings T., Feng Y., Pei W., O’Horo C., Chen J. (2002). Age-dependent decline of DNA repair activity for oxidative lesions in rat brain mitochondria. J. Neurochem..

[161] Daiber A., Oelze M., Wenzel P., Wickramanayake J.M., Schuhmacher S., Jansen T., Lackner K.J., Torzewski M., Munzel T. (2009). Nitrate tolerance as a model of vascular dysfunction: Roles for mitochondrial aldehyde dehydrogenase and mitochondrial oxidative stress. Pharmacol. Rep..

[162] Daiber A., Oelze M., Coldewey M., Bachschmid M., Wenzel P., Sydow K., Wendt M., Kleschyov A.L., Stalleicken D., Ullrich V. (2004). Oxidative stress and mitochondrial aldehyde dehydrogenase activity: A comparison of pentaerythritol tetranitrate with other organic nitrates. Mol. Pharmacol..

[163] Sydow K., Daiber A., Oelze M., Chen Z., August M., Wendt M., Ullrich V., Mulsch A., Schulz E., Keaney J.F. (2004). Central role of mitochondrial aldehyde dehydrogenase and reactive oxygen species in nitroglycerin tolerance and cross-tolerance. J. Clin. Investig..

[164] Esplugues J.V., Rocha M., Nunez C., Bosca I., Ibiza S., Herance J.R., Ortega A., Serrador J.M., D’Ocon P., Victor V.M. (2006). Complex I dysfunction and tolerance to nitroglycerin: An approach based on mitochondrial-targeted antioxidants. Circ. Res..

[165] Wenzel P., Mollnau H., Oelze M., Schulz E., Wickramanayake J.M., Muller J., Schuhmacher S., Hortmann M., Baldus S., Gori T. (2008). First evidence for a crosstalk between mitochondrial and nadph oxidase-derived reactive oxygen species in nitroglycerin-triggered vascular dysfunction. Antioxid. Redox Signal..

[166] Dikalov S.I., Nazarewicz R.R., Bikineyeva A., Hilenski L., Lassegue B., Griendling K.K., Harrison D.G., Dikalova A.E. (2014). Nox2-induced production of mitochondrial superoxide in angiotensin II-mediated endothelial oxidative stress and hypertension. Antioxid. Redox Signal..

[167] Doughan A.K., Harrison D.G., Dikalov S.I. (2008). Molecular mechanisms of angiotensin II-mediated mitochondrial dysfunction: Linking mitochondrial oxidative damage and vascular endothelial dysfunction. Circ. Res..

[168] Nazarewicz R.R., Dikalova A.E., Bikineyeva A., Dikalov S.I. (2013). Nox2 as a potential target of mitochondrial superoxide and its role in endothelial oxidative stress. Am. J. Physiol. Heart Circ. Physiol..

[169] Brandes R.P. (2005). Triggering mitochondrial radical release: A new function for NADPH oxidases. Hypertension.

[170] Kimura S., Zhang G.X., Nishiyama A., Shokoji T., Yao L., Fan Y.Y., Rahman M., Abe Y. (2005). Mitochondria-derived reactive oxygen species and vascular MAP kinases: Comparison of angiotensin II and diazoxide. Hypertension.

[171] Dikalova A.E., Bikineyeva A.T., Budzyn K., Nazarewicz R.R., McCann L., Lewis W., Harrison D.G., Dikalov S.I. (2010). Therapeutic targeting of mitochondrial superoxide in hypertension. Circ. Res..

[172] Fukui T., Ishizaka N., Rajagopalan S., Laursen J.B., Capers Q.T., Taylor W.R., Harrison D.G., de Leon H., Wilcox J.N., Griendling K.K. (1997). p22phox mRNA expression and NADPH oxidase activity are increased in aortas from hypertensive rats. Circ. Res..

[173] Cheresh P., Kim S.J., Tulasiram S., Kamp D.W. (2013). Oxidative stress and pulmonary fibrosis. Biochim. Biophys. Acta.

[174] Nazarewicz R.R., Dikalov S.I. (2013). Mitochondrial ROS in the prohypertensive immune response. Am. J. Physiol. Regul. Integr. Comp. Physiol..

[175] Bender A., Hajieva P., Moosmann B. (2008). Adaptive antioxidant methionine accumulation in respiratory chain complexes explains the use of a deviant genetic code in mitochondria. Proc. Natl. Acad. Sci. USA.

[176] Moskovitz J., Bar-Noy S., Williams W.M., Requena J., Berlett B.S., Stadtman E.R. (2001). Methionine sulfoxide reductase (MsrA) is a regulator of antioxidant defense and lifespan in mammals. Proc. Natl. Acad. Sci. USA.

[177] Stadtman E.R., Moskovitz J., Berlett B.S., Levine R.L. (2002). Cyclic oxidation and reduction of protein methionine residues is an important antioxidant mechanism. Mol. Cell. Biochem..

[178] Taungjaruwinai W.M., Bhawan J., Keady M., Thiele J.J. (2009). Differential expression of the antioxidant repair enzyme methionine sulfoxide reductase (MSRA and MSRB) in human skin. Am. J. Dermatopathol..

[179] Mochin M.T., Underwood K.F., Cooper B., McLenithan J.C., Pierce A.D., Nalvarte C., Arbiser J., Karlsson A.I., Moise A.R., Moskovitz J. (2015). Hyperglycemia and redox status regulate RUNX2 DNA-binding and an angiogenic phenotype in endothelial cells. Microvasc. Res..

[180] Gu H., Chen W., Yin J., Chen S., Zhang J., Gong J. (2013). Methionine sulfoxide reductase A rs10903323 G/A polymorphism is associated with increased risk of coronary artery disease in a chinese population. Clin. Biochem..

[181] Haenold R., Wassef R., Brot N., Neugebauer S., Leipold E., Heinemann S.H., Hoshi T. (2008). Protection of vascular smooth muscle cells by over-expressed methionine sulphoxide reductase A: Role of intracellular localization and substrate availability. Free Radic. Res..

[182] Moosmann B., Behl C. (2008). Mitochondrially encoded cysteine predicts animal lifespan. Aging Cell.

[183] Camici G.G., Cosentino F., Tanner F.C., Luscher T.F. (2008). The role of p66Shc deletion in age-associated arterial dysfunction and disease states. J. Appl. Physiol..

[184] Francia P., delli Gatti C., Bachschmid M., Martin-Padura I., Savoia C., Migliaccio E., Pelicci P.G., Schiavoni M., Luscher T.F., Volpe M. (2004). Deletion of p66Shc gene protects against age-related endothelial dysfunction. Circulation.

[185] Gertz M., Fischer F., Wolters D., Steegborn C. (2008). Activation of the lifespan regulator p66Shc through reversible disulfide bond formation. Proc. Natl. Acad. Sci. USA.

[186] Pinton P., Rimessi A., Marchi S., Orsini F., Migliaccio E., Giorgio M., Contursi C., Minucci S., Mantovani F., Wieckowski M.R. (2007). Protein kinase C β and prolyl isomerase 1 regulate mitochondrial effects of the life-span determinant p66Shc. Science.

[187] Pinton P., Rizzuto R. (2008). P66Shc, oxidative stress and aging: Importing a lifespan determinant into mitochondria. Cell. Cycle.

[188] Rota M., LeCapitaine N., Hosoda T., Boni A., De Angelis A., Padin-Iruegas M.E., Esposito G., Vitale S., Urbanek K., Casarsa C. (2006). Diabetes promotes cardiac stem cell aging and heart failure, which are prevented by deletion of the p66Shc gene. Circ. Res..

[189] Yamamori T., White A.R., Mattagajasingh I., Khanday F.A., Haile A., Qi B., Jeon B.H., Bugayenko A., Kasuno K., Berkowitz D.E. (2005). p66Shc regulates endothelial no production and endothelium-dependent vasorelaxation: Implications for age-associated vascular dysfunction. J. Mol. Cell. Cardiol..

[190] Trinei M., Giorgio M., Cicalese A., Barozzi S., Ventura A., Migliaccio E., Milia E., Padura I.M., Raker V.A., Maccarana M. (2002). A p53-p66Shc signalling pathway controls intracellular redox status, levels of oxidation-damaged DNA and oxidative stress-induced apoptosis. Oncogene.

[191] Di Lisa F., Kaludercic N., Carpi A., Menabo R., Giorgio M. (2009). Mitochondrial pathways for ROS formation and myocardial injury: The relevance of p66Shc and monoamine oxidase. Basic Res. Cardiol..

[192] Spescha R.D., Klohs J., Semerano A., Giacalone G., Derungs R.S., Reiner M.F., Rodriguez Gutierrez D., Mendez-Carmona N., Glanzmann M., Savarese G. (2015). Post-ischaemic silencing of p66Shc reduces ischaemia/reperfusion brain injury and its expression correlates to clinical outcome in stroke. Eur. Heart J..

[193] Moskalev A.A., Aliper A.M., Smit-McBride Z., Buzdin A., Zhavoronkov A. (2014). Genetics and epigenetics of aging and longevity. Cell. Cycle.

[194] Barbot W., Dupressoir A., Lazar V., Heidmann T. (2002). Epigenetic regulation of an IAP retrotransposon in the aging mouse: Progressive demethylation and de-silencing of the element by its repetitive induction. Nucleic Acids Res..

[195] Narita M., Nunez S., Heard E., Narita M., Lin A.W., Hearn S.A., Spector D.L., Hannon G.J., Lowe S.W. (2003). Rb-mediated heterochromatin formation and silencing of E2F target genes during cellular senescence. Cell..

[196] Tsurumi A., Li W.X. (2012). Global heterochromatin loss: A unifying theory of aging?. Epigenetics.

[197] McCauley B.S., Dang W. (2014). Histone methylation and aging: Lessons learned from model systems. Biochim. Biophys. Acta.

[198] Bilsland A.E., Revie J., Keith W. (2013). Microrna and senescence: The senectome, integration and distributed control. Crit. Rev. Oncog..

[199] Boon R.A., Iekushi K., Lechner S., Seeger T., Fischer A., Heydt S., Kaluza D., Treguer K., Carmona G., Bonauer A. (2013). MicroRNA-34a regulates cardiac ageing and function. Nature.

[200] Guarner V., Rubio-Ruiz M.E. (2015). Low-grade systemic inflammation connects aging, metabolic syndrome and cardiovascular disease. Interdiscip. Top. Gerontol..

[201] Howcroft T.K., Campisi J., Louis G.B., Smith M.T., Wise B., Wyss-Coray T., Augustine A.D., McElhaney J.E., Kohanski R., Sierra F. (2013). The role of inflammation in age-related disease. Aging.

[202] Martin-Montalvo A., Mercken E.M., Mitchell S.J., Palacios H.H., Mote P.L., Scheibye-Knudsen M., Gomes A.P., Ward T.M., Minor R.K., Blouin M.J. (2013). Metformin improves healthspan and lifespan in mice. Nat. Commun..

[203] Wilson P.W. (1994). Established risk factors and coronary artery disease: The framingham study. Am. J. Hypertens..

[204] Munzel T., Sinning C., Post F., Warnholtz A., Schulz E. (2008). Pathophysiology, diagnosis and prognostic implications of endothelial dysfunction. Ann. Med..

[205] Munzel T., Gori T., Bruno R.M., Taddei S. (2010). Is oxidative stress a therapeutic target in cardiovascular disease?. Eur. Heart J..

[206] Schachinger V., Britten M.B., Zeiher A.M. (2000). Prognostic impact of coronary vasodilator dysfunction on adverse long-term outcome of coronary heart disease. Circulation.

[207] Gori T., Munzel T. (2011). Oxidative stress and endothelial dysfunction: Therapeutic implications. Ann. Med..

[208] Chen A.F., Chen D.D., Daiber A., Faraci F.M., Li H., Rembold C.M., Laher I. (2012). Free radical biology of the cardiovascular system. Clin. Sci..

[209] Kimura Y., Matsumoto M., Den Y.B., Iwai K., Munehira J., Hattori H., Hoshino T., Yamada K., Kawanishi K., Tsuchiya H. (1999). Impaired endothelial function in hypertensive elderly patients evaluated by high resolution ultrasonography. Can. J. Cardiol..

[210] Wray D.W., Nishiyama S.K., Harris R.A., Zhao J., McDaniel J., Fjeldstad A.S., Witman M.A., Ives S.J., Barrett-O’Keefe Z., Richardson R.S. (2012). Acute reversal of endothelial dysfunction in the elderly after antioxidant consumption. Hypertension.

[211] Jousilahti P., Vartiainen E., Tuomilehto J., Puska P. (1999). Sex, age, cardiovascular risk factors, and coronary heart disease: A prospective follow-up study of 14 786 middle-aged men and women in Finland. Circulation.

[212] Holzer M., Trieb M., Konya V., Wadsack C., Heinemann A., Marsche G. (2013). Aging affects high-density lipoprotein composition and function. Biochim. Biophys. Acta.

[213] Besler C., Heinrich K., Riwanto M., Luscher T.F., Landmesser U. (2010). High-density lipoprotein-mediated anti-atherosclerotic and endothelial-protective effects: A potential novel therapeutic target in cardiovascular disease. Curr. Pharm. Des..

[214] Wu Z., Siuda D., Xia N., Reifenberg G., Daiber A., Munzel T., Forstermann U., Li H. (2015). Maternal treatment of spontaneously hypertensive rats with pentaerythritol tetranitrate reduces blood pressure in female offspring. Hypertension.

[215] Gioscia-Ryan R.A., LaRocca T.J., Sindler A.L., Zigler M.C., Murphy M.P., Seals D.R. (2014). Mitochondria-targeted antioxidant (MitoQ) ameliorates age-related arterial endothelial dysfunction in mice. J. Physiol..

[216] Klipstein-Grobusch K., Geleijnse J.M., den Breeijen J.H., Boeing H., Hofman A., Grobbee D.E., Witteman J.C. (1999). Dietary antioxidants and risk of myocardial infarction in the elderly: The rotterdam study. Am. J. Clin. Nutr..

